# IMproved exercise tolerance in patients with PReserved Ejection fraction by Spironolactone on myocardial fibrosiS in Atrial Fibrillation rationale and design of the IMPRESS-AF randomised controlled trial

**DOI:** 10.1136/bmjopen-2016-012241

**Published:** 2016-10-05

**Authors:** Eduard Shantsila, Ronnie Haynes, Melanie Calvert, James Fisher, Paulus Kirchhof, Paramjit S Gill, Gregory Y H Lip

**Affiliations:** 1 University of Birmingham Institute of Cardiovascular Sciences, City Hospital, Birmingham, UK; 2 Department of Primary Care Clinical Sciences, Institute of Applied Health Research, University of Birmingham, Birmingham, UK; 3 School of Sport, Exercise and Rehabilitation Sciences, University of Birmingham, Birmingham, UK; 4 Institute of Cardiovascular Sciences, University of Birmingham, Birmingham, UK

**Keywords:** atrial fibrillation, heart failure with preserved ejection fraction, spironolactone, exercise tolerance

## Abstract

**Introduction:**

Patients with atrial fibrillation frequently suffer from heart failure with preserved ejection fraction. At present there is no proven therapy to improve physical capacity and quality of life in participants with permanent atrial fibrillation with preserved left ventricular contractility.

**Objective:**

The single-centre IMproved exercise tolerance In heart failure With PReserved Ejection fraction by Spironolactone On myocardial fibrosiS In Atrial Fibrillation (IMPRESS-AF) trial aims to establish whether treatment with spironolactone as compared with placebo improves exercise tolerance (cardiopulmonary exercise testing), quality of life and diastolic function in patients with permanent atrial fibrillation.

**Methods and analysis:**

A total of 250 patients have been randomised in this double-blinded trial for 2-year treatment with 25 mg daily dose of spironolactone or matched placebo. Included participants are 50 years old or older, have permanent atrial fibrillation and ejection fraction >55%. Exclusion criteria include contraindications to spironolactone, poorly controlled hypertension and presence of severe comorbidities with life expectancy <2 years. The primary outcome is improvement in exercise tolerance at 2 years and key secondary outcomes include quality of life (assessed using the EuroQol EQ-5D-5L (EQ-5D) and Minnesota Living with Heart Failure (MLWHF) questionnaires), diastolic function and all-cause hospitalisation.

**Ethics and dissemination:**

The study has been approved by the National Research and Ethics Committee West Midlands—Coventry and Warwickshire (REC reference number 14/WM/1211). The results of the trial will be published in an international peer-reviewed journal.

**Trial registration numbers:**

EudraCT2014-003702-33; NCT02673463; Pre-results.

**Strengths and limitations of this study**

- Double-blinded randomised placebo-controlled study design.
- Accurate assessment of exercise tolerance (the primary outcome) using cardiopulmonary exercise testing.
- Recruitment from primary and secondary care settings to provide a representative population of patients.
- Single-centre study.
- Assessment of effect of the treatment for mortality is beyond the study statistical power.

## Introduction

Heart failure (HF) with preserved ejection fraction (HFpEF) is an emerging problem of modern cardiology, represents about half of all cases of HF, and is very common in individuals with atrial fibrillation (AF).^1–3^ In the Framingham Heart Study, 37% of participants with new AF had HF and presence of AF was strongly related to incident HFpEF (HR 2.34, 95% CI 1.48 to 3.70).^4^ Despite preservation of left ventricular ejection fraction (LVEF), patients with HFpEF have poor quality of life, high morbidity and mortality; largely comparable to HF with reduced LVEF.^5^ Improvements in morbidity and mortality with conventional treatments used in HF with reduced LVEF, however, have not translated to HFpEF.^6^

AF is present in about 40% of participants with HFpEF and is associated with higher N-terminal pro b-type natriuretic peptide (NT-proBNP) levels, risk of death and hospital admission with HF.^7–10^ In the Candesartan in Heart failure-Assessment of Reduction in Mortality and morbidity (CHARM) programme, AF was associated with increased risk of death or hospitalisation for worsening HFpEF (HR 1.72, 95% CI 1.45 to 2.06 for adverse cardiovascular outcomes).^8^

The mechanisms leading to symptoms, morbidity and mortality in patients with HFpEF and AF are poorly understood. Under physiological conditions, left ventricular pressure rapidly decays after systole, allowing low filling pressures and adequate diastolic filling. In HFpEF, the diastolic filling is compromised as a result of aggravation in active and passive relaxation (increased cardiac stiffness).^11^ This ventricular filling abnormality, in turn, reduces cardiac output leads to symptoms of HF.^1^ This theory is supported by interventional experiments and by large population-based studies carried out using a non-invasive approach to measure diastolic stiffness.^12–14^ Furthermore, the elevated filling pressure will increase pressure in the pulmonary system and eventually lead to pulmonary hypertension and pulmonary oedema in acute settings. A stiff ventricle may possess only limited ability to use the Frank-Starling mechanism to increase stroke volume during exercise with increasing heart rates.^15^

While activation of profibrotic pathways is a known response to increased pressure load in the heart, increased production of myocardial collagen and development of fibrosis can also aggravate diastolic dysfunction and ventricular stiffness. Increased myocardial collagen turnover and shift in the balance between matrix metalloproteinases and their inhibitors also favour of excessive myocardial fibrosis.^16^ ^17^

Aldosterone is an important promoter of left ventricular fibrosis.^18^ Mechanisms of aldosterone-mediated cardiac fibrosis include myocardial inflammation, oxidative stress, and cardiomyocyte apoptosis and also direct stimulation of cardiac fibroblasts to produce collagen.^19^ ^20^ Cardiac expression of mineralocorticoid receptors is increased in AF, thus augmenting the genomic effects of aldosterone.^21^

The effectiveness of spironolactone in HFpEF has been tested recently in two clinical trials. The Aldosterone Receptor Blockade in Diastolic Heart Failure (ALDO-DHF) study mainly enrolled participants with hypertensive, another major risk factor for HFpEF.^22^ ^23^ While 92% of the trial patients had hypertension, only 5% of the study population (n=22) had AF at presentation.^22^ ^23^ The Treatment of Preserved Cardiac Function Heart Failure With an Aldosterone Antagonist (TOPCAT) study^24^ ^25^ included a higher proportion of participants with AF (mainly paroxysmal AF). The study defined preserved left ventricular function as LVEF≥45%, thus recruiting a proportion of participants with impaired LVEF according to contemporary definitions (also called ‘HF with intermediate ejection fraction’ by some).^1^ ^26^ Thus, the current evidence on the effectiveness of spironolactone in patients with AF with preserved LVEF on morbidity and quality of life is sparse. We, therefore, plan the IMproved exercise tolerance In heart failure With PReserved Ejection fraction by Spironolactone On myocardial fibrosiS In Atrial Fibrillation (IMPRESS-AF) trial to determine the effects of spironolactone in permanent AF with preserved LVEF.

### Study objectives

The IMPRESS-AF trial aims to establish whether, in participants with permanent AF, treatment with spironolactone as compared with placebo will improve exercise tolerance as a surrogate for cardiovascular mortality/morbidity (primary outcome); and will improve quality of life and diastolic function, as well as reduce the rate of all-cause hospital admissions, and increase rate of spontaneous cardioversion to sinus rhythm (secondary outcomes). The IMPRESS-AF trial will provide evidence on the clinical effectiveness of a readily available treatment in participants with AF with preserved LVEF.

### Study design

The IMPRESS-AF is a double-blinded, randomised, placebo-controlled single-centre trial conducted in Birmingham, UK. The trial aims to recruit 250 participants permanent AF and LVEF>55% from primary and secondary care to be randomised to either spironolactone or placebo. Recruitment of the planned 250 patients was completed on 29 June 2016. The trial protocol was developed following the Standard Protocol Items for Randomized Trials (SPIRIT) statement and the latest patient-reported outcome (PRO)-specific guidance from the International Society for Quality of Life Research (ISOQOL) Best Practice for PROs in trials taskforce.^27–29^ The full protocol is available (see online supplementary appendix 1).

10.1136/bmjopen-2016-012241.supp1 supplementary appendix

#### Eligibility

The main inclusion and exclusion criteria are summarised in table 1. Eligible patients are of male or female gender and age of 50 years or older. Permanent AF is defined by the European Society of Cardiology criteria.^30^ ^31^ All participants have LVEF>55% as established by echocardiography during the screening.^32^ The prospective participants must be able to perform cardiopulmonary exercise testing using a cycling ergometer and complete quality of life questionnaires in English in their native language. For this, an interpreter and translated materials are provided if English is not their spoken language. Average values from 10 consecutive cardiac cycles are calculated to establish LVEF and ratio of peak velocities of early diastolic mitral inflow and peak early tissue Doppler velocity (E/e’). In patients with hypertension, antihypertensive treatment was established before the recruitment and patients with systolic blood pressure more than 160 mm Hg were excluded.

**Table 1:** Key eligibility criteria for IMPRESS-AF

Inclusion criteria	Exclusion criteria
Permanent AF	LVEF<55% (echocardiography)
Age 50 years old or over	Severe systemic illness (life expectancy <2 years)
Ability to understand and complete questionnaires (with or without use of a translater/translated materials)	Severe COPD (eg, requiring home oxygen or chronic oral steroid therapy)
	Severe mitral/aortal valve stenosis/regurgitation
	Significant renal dysfunction (serum creatinine 220 µmol/L or above), anuria, active renal insufficiency, rapidly progressing or severe impairment of renal function, confirmed or suspected renal insufficiency in patients with diabetes/diabetic nephropathy
	Increase in potassium level to >5 mmol/L
	Recent coronary artery bypass graft surgery (within 3 months)
	Use of aldosterone antagonist within 14 days before randomisation
	Use of or potassium sparing diuretic within 14 days before randomisation
	Systolic blood pressure >160 mm Hg
	Addison's disease
	Hypersensitivity to spironolactone or any of the ingredients in the product
	Any participant characteristic that may interfere with adherence to the trial protocol

AF, atrial fibrillation; COPD, chronic obstructive pulmonary disease; IMPRESS-AF, IMproved exercise tolerance In heart failure With PReserved Ejection fraction by Spironolactone On myocardial fibrosiS In Atrial; LVEF, left ventricular ejection fraction.

To improve generalisability, we do not include a requirement for evidence of diastolic dysfunction, as the trial patients would have impaired diastolic function due to AF. The principal exclusion criteria are designed to exclude patients with contraindications to spironolactone or significant comorbidities, which would prevent the prospective participants from completion of the study without relation to the study objectives. All participants will receive current optimised treatment following established clinical guidelines on management of AF, HF and hypertension.^1^

#### Trial setting and identification of participants

The trial is coordinated by Primary Care Research and Clinical Trials Unit (PC-RCTU), University of Birmingham, including coordination of the participant searches, using clinical research network. All patients are seen, investigated and managed in the Research Clinic in the Institute of Cardiovascular Sciences (RC-ICS), City Hospital, Birmingham.

**Figure 1:**
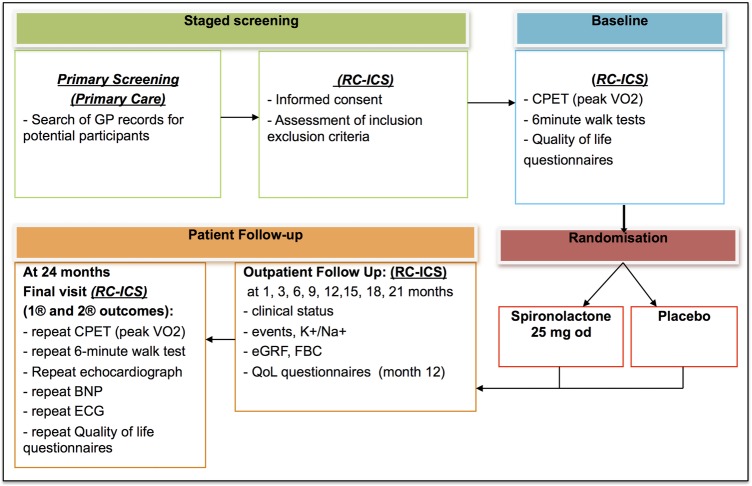
Trial schema. BNP, brain natriuretic peptide; CPET, cardio-pulmonary exercise testing; eGRF, estimated glomerular filtration rate; FBC, full blood count and haematocrit; GP, general practitioner; QoL, quality of life; RC-CCS, Research clinic of the University of Birmingham Institute for Cardiovascular Sciences, City Hospital, Birmingham, UK.

Trial participants have been recruited from primary care AF registers in family practices and outpatient AF clinics in Sandwell and West Birmingham Hospitals Trust, Birmingham. This allowed enrolment of a representative population of patients with AF. At the screening visit to the RC-ICS participants were consented into the study and screened for eligibility. During the baseline visit, the eligible patients undergone cardiopulmonary exercise testing using a cycling ergometer (to measure peak oxygen consumption (VO_2_)), 6 min walk test and complete quality of life questionnaires (validated Minnesota Living with Heart Failure (MLWHF)^33–35^ and EuroQol EQ-5D-5L (EQ-5D)^36^ ^37^ questionnaires). After that, they were randomised into the 2-year trial. The study schema and visit schedule are shown in figure 1 and table 2.

**Table 2:** Timeline of trial procedures alongside the assessments that will be carried out at each stage

			Follow-up								
Visit	Screening	Baseline	Month 1	Month 3	Month 6	Month 9	Month 12	Month 15	Month 18	Month 21	Month 24
		Additional visits will be arranged to reassess potassium levels if patient's blood results show a potassium level of >5.0 mmol/L									
Eligibility check	X	X									
Informed consent	X										
Relevant medical history taken	X										
Concomitant medication	X	X	X	X	X	X	X	X	X	X	X
Standard clinical examination including BP check	X	X	X	X	X	X	X	X	X	X	X
Clinical biochemistry											
Full blood count	X		X	X	X	X	X	X	X	X	X
Renal function, potassium, sodium	X		X	X	X	X	X	X	X	X	X
HbA1c (for diabetics)	X										
Lipid levels	X										
ECG	X										X
Echocardiogram	X										X
Brain natriuretic peptide test	X										X
Randomisation		X									
Dispensing of study drug		X			X		X		X		
Cardiopulmonary exercise testing		X									X
6 min walk test		X									X
Quality of life questionnaires		X					X				X

BP, blood pressure; HbA1c, glycated haemoglobin.

#### Randomisation and blinding

During randomisation (1:1), the participants were first stratified by their baseline peak VO_2_ (two stratification groups; VO_2_≤16 mL/min/kg, and VO_2_>16 mL/min/kg). A secure web-based randomisation system was used for the concealed allocation of a unique investigational medicinal product number to each participant. Trial participants, the trial team in contact with the patient, care providers, outcome assessors and data analysts all remain blinded to the treatment.

Blinding of the trial drug identity took place at the time of packaging and labelling (Catalent Pharma Solutions, UK). Only the database programmer and the Catalent Pharma Solutions can see the investigational medicinal product number list. A sealed copy of the list is kept to the Pharmacy Department at City Hospital (who are independent of the trial, and operate 24 hours a day). In the event of a codebreak situation occurring, the patient will be withdrawn from the trial treatment, as they will become unblinded to their trial drug.

#### Treatment and dosing schedule

Trial participants receive either spironolactone 25 mg once daily or matched placebo. This dose has been shown to improve outcomes in systolic HF, improve diastolic function in HFpEF and to reduce collagen turnover, a marker for fibrotic signalling, in the The Randomized Aldactone Evaluation Study (RALES) population.^38^ The same dose of the spironolactone within 1 year significantly improved diastolic function in participants with HFpEF from the ALDO-DHF trial.^23^

In the case of an increase in potassium level to 5.1–5.5 mmol/L or in the presence of other non-life-threatening side effects (such as gynaecomastia) the trial drug is downtitrated to 25 mg each second day. In such cases, the investigators are advised to reuptitrate the trial medication if the reason for downtitration has resolved.

Drug toxicity will be defined as an increase in potassium level to >5.5 mmol/L. In the case of toxicity or suspected toxicity, the trial medication will be stopped for the duration of the trial, but the patient will be requested to attend the remaining follow-up visits. Blood pressure will be controlled during the duration of the study with particular attention to blood pressure levels after beginning of the study drug and after any changes in antihypertensive agents and their doses.

### Study end points

The *primary* efficacy end point will be the improvement in exercise tolerance at 2 years. This will be assessed by the difference between trial groups in peak VO_2_ on cardiopulmonary exercise testing.

The *secondary* efficacy end point will be the level of improvement in quality of life and diastolic function, and also the improvement the rate of all-cause hospital admissions and spontaneous return the sinus rhythm, with spironolactone. This will be assessed by: (1) improvement in exercise tolerance measured by 6 min walking test (a simple test of exercise performance) at baseline and at 2 years; (2) improvement in quality of life (MLWHF and EQ-5D^36^ ^37^ questionnaires) over the 2-year duration; (3) improvement in left ventricular diastolic function (E/e’ ratio^39–45^ on echocardiography) will be assessed at baseline and at 2 years; (4) improvement in rates of all-cause hospitalisations during 2-year follow-up;^35^ ^36^ (5) spontaneous return to sinus rhythm on ECG after 2 years of treatment. Additionally we will record any cases of major adverse clinical events, such as death from any causes, death from cardiac causes, hospitalisation for cardiac causes, a change in the New York Heart Association (NYHA) class, stroke or systemic thromboembolism.

The study started on the October 2014 and recruitment completed on 29 June 2016. We plan to complete the study by September 2018.

### Statistical considerations

The analysis will follow intention-to-treat principles. The linear mixed-model analysis will be used to compare peak VO_2_ at 2 years between the intervention and the control group. Covariates will be peak baseline VO_2_, age, gender, systolic/diastolic blood pressure and body mass index measured at baseline. General practitioner practices or recruitment centres will be included as random effects.

Secondary analyses will also use linear or non-linear mixed modelling as above but with the dependent variable the secondary end points mentioned in the earlier Trial end points section. Interactions between intervention/control, age and gender will also be included in the mixed modelling analyses to see whether differences in secondary end points between intervention and control participants vary with these two factors. Missing values will be substituted using a multiple imputation procedure. Because of the likelihood of non-normality, the method of Hussain *et al*^46^ will be used.

For the primary outcome, we based our power calculation for peak VO_2_ on the published values of peak VO_2_ in participants with HF (16±5 mL/min/kg).^47^ We anticipate a difference of 2 mL/min/kg in the improvement in peak VO_2_ after 2-year treatment with spironolactone compared with the control group. Published data in HFpEF suggest that such a difference would be clinically relevant and it was factored for the design of the recent ALDO-DHF study of spironolactone in patients with HFpEF, 95% of whom were free from AF.^22^ ^23^ ^48^ Unfortunately, the study by Cicoira *et al*^47^ used for power calculation does not give a SD of the change in peak VO_2_ from the baseline but a similar trial, Edelmann *et al*^49^ provides that statistic (5 mL/min/kg) and also reports a similar magnitude of the effect. We estimate that a sample size of 100 participants in each arm would give the power of at least 80% to detect differences in primary and secondary end points of a magnitude consistent with published results from similar studies. The inclusion of a provision for a 20% drop out rate could potentially lead to powers of near 90% or more if the assumption of a drop rate of 20% were too pessimistic.

### Study funding and management

The IMPRESS-AF trial is funded by the National Institute for Health Research (NIHR), UK. The University of Birmingham is the sponsor of this trial. The day-to-day management of the trial will be coordinated by the Primary Care Research and Clinical Trials Unit (PC-CRTU) at the University of Birmingham, registered by the NIHR as a trials unit. The Trial Management Group will meet at least monthly to ensure implementation of the trial. A Trial Steering Committee has been appointed and will be responsible for overseeing the progress of the trial. An independent Data Monitoring and Ethics Committee will be responsible for the regular monitoring of trial data and it will give advice on whether the accumulated data from the trial, together with the results from other relevant research, justify the continuing recruitment of further participants. The Data Monitoring and Ethics Committee will make confidential recommendations to the Trial Steering Committee as the decision-making committee for the trial.

### Ethics and dissemination of findings

The results of the trial will be published in an international peer-reviewed journal. We hope that the study findings will inform future guidelines for management of HF.

*Registration*: The study is registered with European Union Clinical Trials Register (EudraCT number 2014-003702-33), clinicaltrial.gov (NCT02673463) and has been adopted by the NIHR Clinical Research Network.

## Discussion

AF has a prominent role in prognostication in HF. In a recent large study of 23 644 participants with HF, of which 48.3% had documented AF, the presence of the arrhythmia was associated with higher adjusted rates of ischaemic stroke, hospitalisation for HF, all-cause hospitalisation and death irrespectively whether LVEF was impaired or preserved.^50^ Clinical trials of aldosterone antagonists (RALES, The Eplerenone Post-Acute Myocardial Infarction Heart Failure Efficacy and Survival Study (EPHESUS), The Eplerenone Post-Acute Myocardial Infarction Heart Failure Efficacy and Survival Study (EMPHASIS-HF)) uniformly showed their clinical benefits in systolic HF. However, there is no established treatment for patients with AF with HFpEF.

Activation of aldosterone pathway can contribute to the progression of patients with AF to symptomatic HF despite preserved cardiac contractility, due to the promotion of cardiac fibrosis. Published evidence from AF populations supports the central role of atrial fibrosis in electrical and structural atrial remodelling, and its independent predictive value for the high risk of cerebrovascular events.^51^ ^52^ There is an association between AF and abnormal left ventricular fibrosis, which related to the depressed diastolic function in such participants.^53^

According to a substudy of the RALES trial, the improved survival in participants treated by spironolactone was linked to its ability to reduce serum markers of ongoing fibrosis (type I and III collagen synthesis).^38^ Additionally, aldosterone leads to cardiac invasion by proinflammatory mononuclear cells.^54^ Aldosterone antagonists (ie, spironolactone or eplerenone) ameliorate left ventricular fibrosis in animal models and reduce levels of serum markers of collagen turnover in humans with HFpEF (n=44).^55^ ^56^ In a small, published pilot trial, spironolactone reduced left ventricular fibrosis and improved diastolic function in participants with HFpEF (dilated cardiomyopathy, n=25).^57^

The randomised IMPRESS-AF study should help understanding utility of aldosterone inhibition in permanent AF for prevention of deterioration or improvement in exercise tolerance and quality of life as well as in cardiac diastolic function.

## References

[1] McMurray JJ, Adamopoulos S, Anker SD ESC Guidelines for the diagnosis and treatment of acute and chronic heart failure 2012: the Task Force for the Diagnosis and Treatment of Acute and Chronic Heart Failure 2012 of the European Society of Cardiology. Developed in collaboration with the Heart Failure Association (HFA) of the ESC. Eur Heart J 2012;33:1787–847. 10.1093/eurheartj/ehs104 22611136

[2] Tsang TS, Barnes ME, Gersh BJ Risks for atrial fibrillation and congestive heart failure in patients >/=65 years of age with abnormal left ventricular diastolic relaxation. Am J Cardiol 2004;93:54–8. 10.1016/j.amjcard.2003.09.012 14697466

[3] Tsang TS, Gersh BJ, Appleton CP Left ventricular diastolic dysfunction as a predictor of the first diagnosed nonvalvular atrial fibrillation in 840 elderly men and women. J Am Coll Cardiol 2002;40:1636–44. 10.1016/S0735-1097(02)02373-2 12427417

[4] Santhanakrishnan R, Wang N, Larson MG Atrial fibrillation begets heart failure and vice versa: temporal associations and differences in preserved vs. reduced ejection fraction. Circulation 2016;133:484–92 10.1161/CIRCULATIONAHA.115.018614. 26746177 PMC4738087

[5] Fonarow GC, Stough WG, Abraham WT Characteristics, treatments, and outcomes of patients with preserved systolic function hospitalized for heart failure: a report from the OPTIMIZE-HF Registry. J Am Coll Cardiol 2007;50:768–77. 10.1016/j.jacc.2007.04.064 17707182

[6] Paulus WJ, van Ballegoij JJ Treatment of heart failure with normal ejection fraction: an inconvenient truth! J Am Coll Cardiol 2010;55:526–37. 10.1016/j.jacc.2009.06.067 20152557

[7] Linssen GC, Rienstra M, Jaarsma T Clinical and prognostic effects of atrial fibrillation in heart failure patients with reduced and preserved left ventricular ejection fraction. Eur J Heart Fail 2011;13:1111–20. 10.1093/eurjhf/hfr066 21642293

[8] Olsson LG, Swedberg K, Ducharme A Atrial fibrillation and risk of clinical events in chronic heart failure with and without left ventricular systolic dysfunction: results from the Candesartan in Heart failure-Assessment of Reduction in Mortality and morbidity (CHARM) program. J Am Coll Cardiol 2006;47:1997–2004. 10.1016/j.jacc.2006.01.060 16697316

[9] McKelvie RS, Komajda M, McMurray J Baseline plasma NT-proBNP and clinical characteristics: results from the irbesartan in heart failure with preserved ejection fraction trial. J Card Fail 2010;16:128–34. 10.1016/j.cardfail.2009.09.007 20142024

[10] Fung JW, Sanderson JE, Yip GW Impact of atrial fibrillation in heart failure with normal ejection fraction: a clinical and echocardiographic study. J Card Fail 2007;13:649–55. 10.1016/j.cardfail.2007.04.014 17923357

[11] Zile MR, Baicu CF, Gaasch WH Diastolic heart failure—abnormalities in active relaxation and passive stiffness of the left ventricle. N Engl J Med 2004;350:1953–9. 10.1056/NEJMoa032566 15128895

[12] Redfield MM, Jacobsen SJ, Borlaug BA Age- and gender-related ventricular-vascular stiffening: a community-based study. Circulation 2005;112:2254–62. 10.1161/CIRCULATIONAHA.105.541078 16203909

[13] Lam CS, Roger VL, Rodeheffer RJ Cardiac structure and ventricular-vascular function in persons with heart failure and preserved ejection fraction from Olmsted County, Minnesota. Circulation 2007;115:1982–90. 10.1161/CIRCULATIONAHA.106.659763 17404159 PMC2001291

[14] Westermann D, Kasner M, Steendijk P Role of left ventricular stiffness in heart failure with normal ejection fraction. Circulation 2008;117:2051–60. 10.1161/CIRCULATIONAHA.107.716886 18413502

[15] Kitzman DW, Higginbotham MB, Cobb FR Exercise intolerance in patients with heart failure and preserved left ventricular systolic function: failure of the Frank-Starling mechanism. J Am Coll Cardiol 1991;17:1065–72. 10.1016/0735-1097(91)90832-T 2007704

[16] Ahmed SH, Clark LL, Pennington WR Matrix metalloproteinases/tissue inhibitors of metalloproteinases: relationship between changes in proteolytic determinants of matrix composition and structural, functional, and clinical manifestations of hypertensive heart disease. Circulation 2006;113:2089–96. 10.1161/CIRCULATIONAHA.105.573865 16636176

[17] Martos R, Baugh J, Ledwidge M Diastolic heart failure: evidence of increased myocardial collagen turnover linked to diastolic dysfunction. Circulation 2007;115:888–95. 10.1161/CIRCULATIONAHA.106.638569 17283265

[18] Weber KT Aldosterone in congestive heart failure. N Engl J Med 2001;345:1689–97. 10.1056/NEJMra000050 11759649

[19] Burniston JG, Saini A, Tan LB Aldosterone induces myocyte apoptosis in the heart and skeletal muscles of rats in vivo. J Mol Cell Cardiol 2005;39:395–9. 10.1016/j.yjmcc.2005.04.001 15907929

[20] Brilla CG, Zhou G, Matsubara L Collagen metabolism in cultured adult rat cardiac fibroblasts: response to angiotensin II and aldosterone. J Mol Cell Cardiol 1994;26:809–20. 10.1006/jmcc.1994.1098 7966349

[21] Tsai CT, Chiang FT, Tseng CD Increased expression of mineralocorticoid receptor in human atrial fibrillation and a cellular model of atrial fibrillation. J Am Coll Cardiol 2010;55:758–70. 10.1016/j.jacc.2009.09.045 20170814

[22] Edelmann F, Schmidt AG, Gelbrich G Rationale and design of the ‘aldosterone receptor blockade in diastolic heart failure’ trial: a double-blind, randomized, placebo-controlled, parallel group study to determine the effects of spironolactone on exercise capacity and diastolic function in patients with symptomatic diastolic heart failure (Aldo-DHF). Eur J Heart Fail 2010;12:874–82. 10.1093/eurjhf/hfq087 20538867

[23] Edelmann F, Wachter R, Schmidt AG Effect of spironolactone on diastolic function and exercise capacity in patients with heart failure with preserved ejection fraction: the Aldo-DHF randomized controlled trial. JAMA 2013;309:781–91. 10.1001/jama.2013.905 23443441

[24] Shah SJ, Heitner JF, Sweitzer NK Baseline characteristics of patients in the treatment of preserved cardiac function heart failure with an aldosterone antagonist trial. Circ Heart Fail 2013;6:184–92. 10.1161/CIRCHEARTFAILURE.112.972794 23258572 PMC3605409

[25] Desai AS, Lewis EF, Li R Rationale and design of the treatment of preserved cardiac function heart failure with an aldosterone antagonist trial: a randomized, controlled study of spironolactone in patients with symptomatic heart failure and preserved ejection fraction. Am Heart J 2011;162:966–72.e10. 10.1016/j.ahj.2011.09.007 22137068

[26] Mahadevan G, Davis RC, Frenneaux MP Left ventricular ejection fraction: are the revised cut-off points for defining systolic dysfunction sufficiently evidence based? Heart 2008;94:426–8. 10.1136/hrt.2007.123877 18347374

[27] Kyte D, Duffy H, Fletcher B Systematic evaluation of the patient-reported outcome (PRO) content of clinical trial protocols. PLoS ONE 2014;9:e110229 10.1371/journal.pone.0110229 25333349 PMC4198237

[28] Calvert M, Kyte D, Duffy H Patient-reported outcome (PRO) assessment in clinical trials: a systematic review of guidance for trial protocol writers. PLoS ONE 2014;9:e110216 10.1371/journal.pone.0110216 25333995 PMC4198295

[29] Calvert M, Kyte D, von Hildebrand M Putting patients at the heart of health-care research. Lancet 2015;385:1073–4. 10.1016/S0140-6736(15)60599-2 25797557

[30] Camm AJ, Lip GY, De Caterina R 2012 focused update of the ESC Guidelines for the management of atrial fibrillation: an update of the 2010 ESC Guidelines for the management of atrial fibrillation. Developed with the special contribution of the European Heart Rhythm Association. Eur Heart J 2012;33:2719–47. 22922413 10.1093/eurheartj/ehs253

[31] Camm AJ, Kirchhof P, Lip GY, European Heart Rhythm A, European Association for Cardio-Thoracic S. Guidelines for the management of atrial fibrillation: the Task Force for the Management of Atrial Fibrillation of the European Society of Cardiology (ESC). Eur Heart J 2010;31:2369–429. 10.1093/eurheartj/ehq278 20802247

[32] Lang RM, Bierig M, Devereux RB Recommendations for chamber quantification. Eur J Echocardiogr 2006;7:79–108. 10.1016/j.euje.2005.12.014 16458610

[33] Rector TS, Carson PE, Anand IS Assessment of long-term effects of irbesartan on heart failure with preserved ejection fraction as measured by the Minnesota living with heart failure questionnaire in the irbesartan in heart failure with preserved systolic function (I-PRESERVE) trial. Circ Heart Fail 2012;5:217–25. 10.1161/CIRCHEARTFAILURE.111.964221 22267751

[34] Rector TS, Kubo SH, Cohn JN Validity of the Minnesota Living with Heart Failure questionnaire as a measure of therapeutic response to enalapril or placebo. Am J Cardiol 1993;71:1106–7. 10.1016/0002-9149(93)90582-W 8475878

[35] Rector TS, Cohn JN Assessment of patient outcome with the Minnesota Living with Heart Failure questionnaire: reliability and validity during a randomized, double-blind, placebo-controlled trial of pimobendan. Pimobendan Multicenter Research Group. Am Heart J 1992;124:1017–25. 10.1016/0002-8703(92)90986-6 1529875

[36] Brooks R EuroQol: the current state of play. Health Policy 1996;37:53–72. 10.1016/0168-8510(96)00822-6 10158943

[37] Rabin R, de Charro F EQ-5D: a measure of health status from the EuroQol Group. Ann Med 2001;33:337–43. 10.3109/07853890109002087 11491192

[38] Zannad F, Alla F, Dousset B Limitation of excessive extracellular matrix turnover may contribute to survival benefit of spironolactone therapy in patients with congestive heart failure: insights from the randomized aldactone evaluation study (RALES). Rales Investigators. Circulation 2000;102:2700–6. 10.1161/01.CIR.102.22.2700 11094035

[39] Sohn DW, Song JM, Zo JH Mitral annulus velocity in the evaluation of left ventricular diastolic function in atrial fibrillation. J Am Soc Echocardiogr 1999;12:927–31. 10552353 10.1016/s0894-7317(99)70145-8

[40] Kusunose K, Yamada H, Nishio S Clinical utility of single-beat E/e’ obtained by simultaneous recording of flow and tissue Doppler velocities in atrial fibrillation with preserved systolic function. JACC Cardiovasc Imaging 2009;2:1147–56. 10.1016/j.jcmg.2009.05.013 19833302

[41] Aljaroudi W, Alraies MC, Halley C Impact of progression of diastolic dysfunction on mortality in patients with normal ejection fraction. Circulation 2012;125:782–8. 10.1161/CIRCULATIONAHA.111.066423 22261198

[42] Nagueh SF, Kopelen HA, Quinones MA Assessment of left ventricular filling pressures by Doppler in the presence of atrial fibrillation. Circulation 1996;94:2138–45. 8901664 10.1161/01.cir.94.9.2138

[43] Temporelli PL, Scapellato F, Corrà U Estimation of pulmonary wedge pressure by transmitral Doppler in patients with chronic heart failure and atrial fibrillation. Am J Cardiol 1999;83:724–7. 10080426 10.1016/s0002-9149(98)00978-3

[44] Chirillo F, Brunazzi MC, Barbiero M Estimating mean pulmonary wedge pressure in patients with chronic atrial fibrillation from transthoracic Doppler indexes of mitral and pulmonary venous flow velocity. J Am Coll Cardiol 1997;30:19–26. 9207616 10.1016/s0735-1097(97)00130-7

[45] Nagueh SF, Smiseth OA, Appleton CP Recommendations for the evaluation of left ventricular diastolic function by echocardiography: an update from the American Society of Echocardiography and the European Association of Cardiovascular Imaging. J Am Soc Echocardiogr 2016;29:277–314. 10.1016/j.echo.2016.01.011 27037982

[46] Hussain S, Mohammed M, Haque M A simple method to ensure plausible multiple imputation for continuous multivariate data. Commun Stat Simulation Comput 2010;39:1779–84. 10.1080/03610918.2010.518267

[47] Cicoira M, Zanolla L, Rossi A Long-term, dose-dependent effects of spironolactone on left ventricular function and exercise tolerance in patients with chronic heart failure. J Am Coll Cardiol 2002;40:304–10. 10.1016/S0735-1097(02)01965-4 12106936

[48] Shafiq A, Brawner CA, Aldred HA Prognostic value of cardiopulmonary exercise testing in heart failure with preserved ejection fraction. The Henry Ford HospITal CardioPulmonary EXercise Testing (FIT-CPX) project. Am Heart J 2016;174:167–72. 10.1016/j.ahj.2015.12.020 26995385 PMC4804356

[49] Edelmann F, Gelbrich G, Düngen HD Exercise training improves exercise capacity and diastolic function in patients with heart failure with preserved ejection fraction: results of the Ex-DHF (Exercise training in Diastolic Heart Failure) pilot study. J Am Coll Cardiol 2011;58:1780–91. 10.1016/j.jacc.2011.06.054 21996391

[50] McManus DD, Hsu G, Sung SH Atrial fibrillation and outcomes in heart failure with preserved versus reduced left ventricular ejection fraction. J Am Heart Assoc 2013;2:e005694 10.1161/JAHA.112.005694 23525446 PMC3603249

[51] Schotten U, Verheule S, Kirchhof P Pathophysiological mechanisms of atrial fibrillation: a translational appraisal. Physiol Rev 2011;91:265–325. 10.1152/physrev.00031.2009 21248168

[52] Daccarett M, Badger TJ, Akoum N Association of left atrial fibrosis detected by delayed-enhancement magnetic resonance imaging and the risk of stroke in patients with atrial fibrillation. J Am Coll Cardiol 2011;57:831–8. 10.1016/j.jacc.2010.09.049 21310320 PMC3124509

[53] Shantsila E, Shantsila A, Blann AD Left ventricular fibrosis in atrial fibrillation. Am J Cardiol 2013;111:996–1001. 10.1016/j.amjcard.2012.12.005 23332595

[54] Weber KT The proinflammatory heart failure phenotype: a case of integrative physiology. Am J Med Sci 2005;330:219–26. 10.1097/00000441-200511000-00004 16284481

[55] Endemann DH, Touyz RM, Iglarz M Eplerenone prevents salt-induced vascular remodeling and cardiac fibrosis in stroke-prone spontaneously hypertensive rats. Hypertension 2004;43:1252–7. 10.1161/01.HYP.0000128031.31572.a3 15117913

[56] Deswal A, Richardson P, Bozkurt B Results of the Randomized Aldosterone Antagonism in Heart Failure With Preserved Ejection Fraction Trial (RAAM-PEF). J Card Fail 2011;17:634–42. 10.1016/j.cardfail.2011.04.007 21807324

[57] Izawa H, Murohara T, Nagata K Mineralocorticoid receptor antagonism ameliorates left ventricular diastolic dysfunction and myocardial fibrosis in mildly symptomatic patients with idiopathic dilated cardiomyopathy: a pilot study. Circulation 2005;112:2940–5. 10.1161/CIRCULATIONAHA.105.571653 16275882

