# Supplementary material for: IMproved exercise tolerance in patients with PReserved Ejection fraction by Spironolactone on myocardial fibrosiS in Atrial Fibrillation rationale and design of the IMPRESS-AF randomised controlled trial
Source: BMJ Open. 2016 Oct 5;6(10):e012241. doi: 10.1136/bmjopen-2016-012241 (PMC5073497; doi:10.1136/bmjopen-2016-012241)
Supplement: supplementary appendix [file bmjopen-2016-012241supp_appendix.pdf]

TRIAL PROTOCOL

IMPRESS - AF

Improved exercise tolerance in participants with **PR**eserved **E**jection fraction by **S**pironolactone on myocardial fibrosi**s** in **A**trial **F**ibrillation

Version 5.0

12th October 2015

Protocol development

Protocol Amendments

The following amendments and/or administrative changes have been made to this protocol since the implementation of the first approved version

Amendment number	Date of amendment	Protocol version number	Type of amendment	Summary of amendment
AM02	*10/03/2015*	*3.0*	*Substantial*	*Change in blood tests*<br>*Change in drug manufacturer*<br>*Removal of GP checklist*<br>*Addition of rebooking tests*<br>*Clarification of randomisation and blinding*
AM03	*01/05/2015*	*4.0*	*Substantial*	*Inclusion in the trial is no longer conditional on the patient having normal BNP levels (<100pg/mL).*<br>*Clarification of personnel designated as CI and PI.*<br>*Trial Management Group to meet regularly rather than weekly (section 14.3).*<br>*Amendment of section 6.3 (Blinding) to clarify that individual sealed codebreak envelopes will be used, rather than a master list.*<br>*Change of trial statistician due to retirement of original person.*<br>*Pharmacy to audit drug storage facility annually, rather than six-monthly, in line with internal NHS policy (section 7.1.4).*<br>*Non-responders to invitation from Primary Care to be sent a reminder letter (Section 4.1 and 8.1).*<br>*Minor corrections to grammar and spelling.*
AM04	*12/10/2015*	*5.0*	*Substantial*	*A mechanistic subanalysis of impact of biomarkers of fibrosis and hemostasis in the study population (Appendix 1)*<br>*To include a timeline for visit*

				windows.<br>*The Ability to understand questionnaires being removed as an inclusion criteria and added as am exclusion criteria.(section 4.1)*

Protocol Sign Off

CI Signature	
This protocol has been approved by:	
Trial Name:	IMPRESS-AF
Protocol Version Number:	Version: 4.0
Protocol Version Date:	20/05/2015
CI Name:	Professor Gregory YH Lip
Trial Role:	Chief Investigator
Signature and date:	20/05/2015
Sponsor statement: By signing the IRAS form for this trial, University of Birmingham, acting as sponsor of this trial confirm approval of this protocol.	

Reference Numbers			
EudraCT number	2014-003702-33	ISRCTN number	ISRCTN10259346
Sponsor number	RG_14-150	REC number	14/WM/1211

Administrative Information

Sponsor
**University of Birmingham**<br>IMPRESS-AF C/O PC-CRTU<br>Primary Care Clinical Research Trials Unit<br>Primary Care Clinical Sciences<br>University of Birmingham<br>Edgbaston<br>Birmingham<br>B15 2TT

Chief-investigator	
Professor Gregory YH Lip	*Consultant Cardiologist & Professor of Cardiovascular Medicine,*<br>*Director - Haemostasis Thrombosis & Vascular Biology Unit*

Co-investigator (s)	
Professor Paulus Kirchhof	*Chair in Cardiovascular Medicine*
Dr Eduard Shantsila (PI)	*Clinical Postdoctoral Research Fellow in Cardiology*
Dr Paramjit S Gill	*Reader in Primary Care Research*
Dr Melanie Calvert	*Professor of Outcomes Methodology*
Dr James Fisher	*Senior Lecturer in Exercise Physiology*
Dr Sayeed Haque	*Medical Statistician*
Dr Mustafa Abul	Patient representative

Trial Office Contact Details	
IMPRESS-AF	IMPRESS-AF Trial Team
Primary Care Clinical Research and Trials Unit<br>Primary Care Clinical Sciences<br>University of Birmingham<br>Edgbaston<br>Birmingham<br>B15 2TT	Tel. 0121 414 7187<br>Fax. 0121 414 3050<br><br>Email: pccrtu@contacts.bham.ac.uk

TRIAL SUMMARY

Title

Improved exercise tolerance in participants with PReserved Ejection fraction by Spironolactone on myocardial fibrosis in Atrial Fibrillation **(IMPRESS-AF)**

Trial Design

This is a double-blind, 2 year randomised, placebo-controlled single centre trial, recruiting 250 participants from primary and secondary care

Objectives

This trial aims to determine whether the aldosterone antagonist, spironolactone improves exercise tolerance and quality of life compared to placebo (both added to the current guideline-recommended optimised care) in symptomatic participants with permanent Atrial Fibrillation (AF).

Participant Population and Sample Size

Participant sample size will be 250 participants recruited from primary and secondary care.

Outcome Measures

Primary outcome:

- Improvement in exercise tolerance at 2 years assessed by difference between the study groups in peak VO2 on CPET

Secondary outcomes:

- Improvement in exercise tolerance measured by 6-minute walking test (a simple and readily available in clinical practice tool for assessment of exercise performance);
- Improvement in quality of life assessed using the validated Minnesota Living with Heart Failure (MLWHF) and [EQ-5D] questionnaires self-completed by patients;
- Improvement in LV diastolic dysfunction (E/E' ratio);
- Rate of all-cause hospitalisations (which will include HF-related hospitalisations).
- Sinus rhythm at 2 year follow up

Additionally we will record any cases of major adverse clinical events, such as death from any causes, death from cardiac causes, hospitalization for cardiac causes, a change in the NYHA class, stroke or systemic thromboembolism.

Eligibility Criteria

Inclusion criteria

- Age 50 years old and over
- Permanent AF as defined by the European Society of Cardiology (ESC) criteria

Exclusion criteria

- Severe systemic illness with life expectancy of <2 years from screening
- Left ventricular ejection fraction (LVEF) <55% (echocardiography)
- Severe *chronic obstructive pulmonary disease (COPD*) (e.g., requiring home oxygen or chronic oral steroid therapy)
- Severe mitral/aortal valve stenosis/regurgitation
- Significant renal dysfunction (serum creatinine 220 µmol/L or above), anuria, active renal insufficiency, rapidly progressing or severe impairment of renal function, confirmed or suspected renal insufficiency in diabetic patients/ diabetic nephropathy
- Increase in potassium level to > 5mmol/L)
- Recent coronary artery bypass graft surgery (within 3 months)
- Use of aldosterone antagonist within 14 days before randomisation
- Use of a potassium sparing diuretic within 14 days before randomisation
- Systolic blood pressure >160 mm Hg
- Addison's disease
- Hypersensitivity to spironolactone or any of the ingredients in the product
- Any participant characteristic that may interfere with adherence to the trial protocol
- In-ability to understand and complete questionnaires (with or without use of a translator/translated materials).

Treatment Allocation

Trial participants will be randomised to receive either Spironolactone or matched placebo, 25milligrams tablet to be taken once a day for 24 months.

Trial Schema

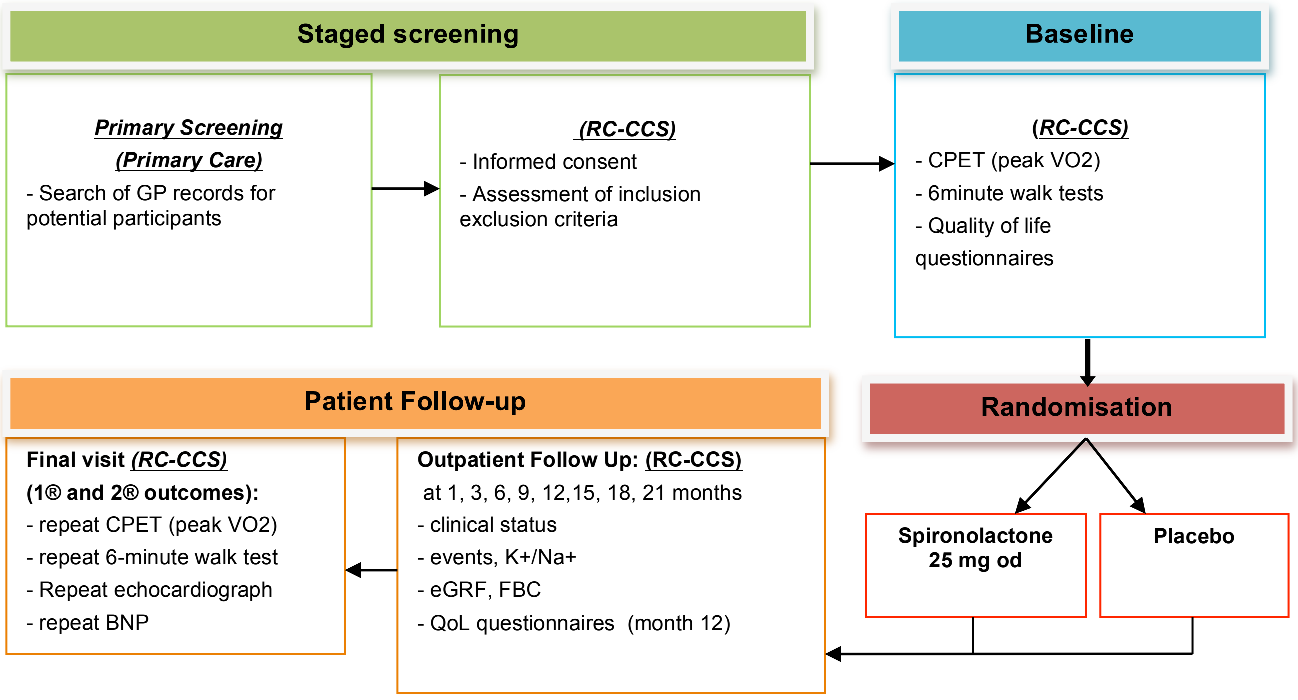

RC-CCS: Research clinic of the University of Birmingham Centre for Cardiovascular Sciences, City Hospital

BNP, brain natriuretic peptide, eGRF, calculated glomerular filtration rate, FBC, full blood count and haematocrit

TABLE OF CONTENTS

1. Background and Rationale ... 12
1.1. Background ... 12
1.2. Trial Rationale ... 15
2. Aims, Objectives and Outcome Measures ... 17
2.1 Aims and Objectives ... 17
2.2 Outcome Measures ... 17
3. Trial Design ... 18
3.1 Trial Design ... 18
3.2 Trial Setting ... 18
4. Trial population ... 19
4.1 Identification of participants ... 19
4.2 Eligibility ... 19
4.1.1 Inclusion Criteria ... 19
4.1.2 Exclusion Criteria ... 19
5. Consent ... 21
6. Enrolment and Randomisation ... 22
6.1 Enrolment ... 22
6.2 Randomisation ... 22
6.3 Blinding ... 23
7. Trial Treatment / intervention ... 24
7.1 Treatment Supply and Storage ... 24
7.1.1 Treatment Supplies ... 24
7.1.2 Packaging and Labelling ... 24
7.1.3 Drug Storage ... 24
7.1.4 Dispensing of treatment ... 25
7.2 Treatment and Dosing Schedule ... 25
7.3 Drug Interaction or Contraindications ... 25
7.4 Accountability Procedures ... 25
7.5 Treatment Modification ... 25
7.6 Concomitant medications ... 25
8. Trial procedures and assessments ... 27
8.1 Summary of assessments ... 28
8.2 Trial Procedures ... 29
8.3 Schedule of Assessments ... 33
8.4 Participant Withdrawal ... 34
9. Adverse Event Reporting ... 35
9.1 Reporting Requirements ... 35

9.2 Adverse Events (AE) ... 35
9.3 Serious Adverse Events (SAE) ... 35
9.3.1 Events that do not require reporting on a Serious Adverse Event Form ... 35
9.3.2 Monitoring pregnancies for potential Serious Adverse Events ... 35
9.4 Reporting period ... 35
9.5 Reporting Procedure – At Site ... 35
9.5.1 Adverse Events ... 35
9.5.2 Serious Adverse Events ... 36
9.5.3 Provision of follow-up information ... 36
9.6 Reporting Procedure – PC-CRTU ... 36
9.6.1 Provision of follow-up information ... 36
9.7 Reporting to the Competent Authority and main Research Ethics Committee ... 36
9.7.1 Suspected Unexpected Serious Adverse Reactions ... 36
9.7.2 Serious Adverse Reactions ... 37
9.7.3 Adverse Events ... 37
9.7.4 Other safety issues identified during the course of the trial ... 37
9.8 Investigators ... 37
9.9 Data Monitoring and Ethics Committee ... 37
10. Data Handling and Record Keeping ... 39
10.1 Source Data ... 39
10.2 CRF and QoL questionnaire Completion ... 39
10.3 Data Management ... 39
10.4 Archiving ... 40
11. Quality control and quality assurance ... 41
11.1 Site Set-up and Initiation ... 41
11.2 Monitoring ... 41
11.2.1 Onsite Monitoring ... 41
11.2.2 Central Monitoring ... 41
11.3 Audit and Inspection ... 42
11.4 Notification of Serious Breaches ... 42
12. End of Trial Definition ... 44
13. Statistical Considerations ... 45
13.1 Analysis of Outcome Measures ... 45
13.1.1 Power Calculations ... 46
14. Trial Organisational Structure ... 48
14.1 Sponsor ... 48
14.2 Coordinating Centre ... 48
14.3 Trial Management Group ... 48

14.3.1 Trial Steering Committee 49
14.3.2 Data Monitoring and Ethics Committee 49
14.4 Finance 49
15. Ethical Considerations 49
16. Confidentiality and Data Protection 51
17. Insurance and Indemnity 52
18. Publication Policy 53
19. Reference List 54
20. ABBREVIATIONS AND DEFINITIONS: 60

1. BACKGROUND AND RATIONALE

1.1. Background

Heart failure (HF) with preserved ejection fraction (HFpEF) is an emerging problem of modern cardiology and represents about half of all cases of HF and it is very common in participants with atrial fibrillation (AF).[1-3] Despite preservation of left ventricular (LV) ejection fraction (EF), morbidity, quality of life (QoL) and mortality are generally similarly poor to those in HF with reduced EF.[4] Furthermore, improvements in morbidity and mortality with conventional treatments used in HF with reduced EF have not translated to HFpEF.[5] Identification of therapies that can improve both QoL and survival is therefore a key priority in the management of HFpEF.

In our local epidemiological screening study (SAFE, funded by the NHS HTA programme), the prevalence of AF was 4.7% in general practice in people aged 65 years or more.[6, 7] The prevalence of AF roughly doubles with each advancing decade of age, being rare below the age of 50 years; showing figures of 0.5% at age 50–59 years and rising to almost 10% at age 80–89 years.[8] A third of AF participants suffer symptomatic HF, of which most have HFpEF.[9] This puts participants with AF under very high risk of developing of HF irrespectively of the status of their systolic function. Accordingly, the targeted participants represent about 3.0% of the UK population 65 years and older and about 1.6% of the population aged 50 years and older (based on the West Birmingham AF project).[10] AF is present in 3–6% of acute medical admissions in the UK.[11, 12]

HF has a poor prognosis. Once HF develops, 30-40% of participants diagnosed with HF die within a year, and thereafter premature mortality continues at a lower rate of 8-10% per year.[13, 14] NICE guidelines on diagnosis and management of chronic HF highlight that *'HF accounts for a total of 1 million inparticipant bed days – 2% of all NHS inparticipant bed-days – and 5% of all emergency medical admissions to hospital'* and that *'As well as NHS costs, heart failure also places a burden on other agencies such as social services and the benefits system, and of course on the participants with heart failure and their families and caregivers'*.[15]

AF is present in about 40% of participants with HFpEF and is independently associated with higher NT-proBNP levels, risk of death and hospitalisation with HF.[16-19] Data from the Candesartan in Heart failure-Assessment of Reduction in Mortality and morbidity (CHARM) program which studied 7,599 participants with symptomatic HF, demonstrated that AF was associated with increased risk of death or hospitalisation for worsening HFpEF.[17] In a recent large study of 23,644 participants with HF, of which 48.3% had documented AF the presence of the arrhythmia was associated with higher adjusted rates of ischemic stroke, hospitalization for HF, all-cause hospitalization, and death irrespectively whether LVEF was impaired or preserved.[20]

High heart rate, loss of atrial systole and irregular cardiac cycle length contribute to the development of HF symptoms in AF participants with preserved cardiac contractility. However, exclusion of participants with AF from recent pathophysiology studies of HFpEF means that there are limited data on the impact of specific treatments aiming to prevent and manage HFpEF pathogenesis in AF.[21, 22] Hence, participants with AF, including those with HFpEF are very commonly encountered in the NHS. Current management of participants with permanent AF is focused on heart rate control in parallel to treatment of other background pathology. Their management is currently not sufficiently guided by either evidence or pathophysiological understanding of disease mechanisms.

The mechanisms leading to development of symptoms of HFpEF in AF despite optimal current treatment AF are poorly understood. Under physiological conditions, left ventricular (LV) pressure rapidly decays after systole, allowing low filling pressures and adequate diastolic filling. In HFpEF, diastolic filling is compromised as a result of aggravation in active and/or passive relaxation

(increased cardiac stiffness).[21] This ventricular filling abnormality in turn reduces cardiac output and is believed to be the main driver of HF symptoms in HFpEF participants.[23] This is supported by both interventional experiments and by large population-based studies carried out using a non-invasive approach to measure diastolic stiffness.[22, 24, 25] Furthermore, elevated filling pressure will increase pressure in the pulmonary system and eventually lead to pulmonary oedema. It has been considered that a stiff ventricle may possess only limited ability to use the Frank-Starling mechanism to increase stroke volume during exercise with increasing heart rates.[26]

While activation of profibrotic pathways is a known response to increased pressure load in the heart, increased production of myocardial collagen and development of fibrosis might also play a key role in diastolic dysfunction and increased cardiac stiffness. This process is linked to an increased myocardial collagen turnover and shift in the balance between matrix metalloproteinases and their inhibitors in favour of excessive myocardial fibrosis.[27, 28] Published evidence from AF populations supports central role of atrial fibrosis in electrical and structural atrial remodelling, and its independent predictive value for the high risk of cerebrovascular events.[29, 30] Our recently published work also indicates association of AF with abnormal LV fibrosis, which was linked to depressed diastolic function in such participants.[31] Together these data suggest that abnormal activation of profibrotic pathways is common in participants with AF and can be important factor for development of HF symptoms despite preserved LV systolic function.

Aldosterone and cardiac fibrosis

Aldosterone is an important promoter of LV fibrosis.[32] Mechanisms of aldosterone-mediated cardiac fibrosis include myocardial inflammation, oxidative stress, and cardiomyocyte apoptosis and also direct stimulation of cardiac fibroblasts to produce collagen.[33, 34] Clinical trials of aldosterone antagonists (RALES, EPHESUS, EMPHASIS-HF) uniformly showed their clinical benefits in systolic HF. Of note, according to a substudy of the RALES trial, the improved survival in participants treated by spironolactone was linked to its ability to reduce serum markers of ongoing fibrosis (type I and III collagen synthesis).[35] Additionally aldosterone leads to cardiac invasion by proinflammatory mononuclear cells.[36]

Cardiac expression of mineralocorticoid receptors is increased in AF, thus augmenting the genomic effects of aldosterone.[37] Aldosterone antagonists (i.e., spironolactone or eplerenone) ameliorate LV fibrosis in animal models and reduce levels of serum markers of collagen turnover in humans with HFpEF (n=44).[38, 39] In a small, published pilot trial, spironolactone reduced LV fibrosis and improved diastolic function in participants with HFpEF (dilated cardiomyopathy, n=25).[40]

In a previous randomised clinical trial on 102 participants with chronic kidney disease with normal cardiac contractility cardiac diastolic function was significantly improved over 40 weeks of treatment by 25 mg daily of spironolactone vs. placebo.[41, 42] Aldosterone inhibition with spironolactone significantly improves diastolic function and reduces cardiovascular stiffness in participants with chronic kidney disease.[41, 42] Recently, the same dose of spironolactone within 1 year significantly improved diastolic function in participants with HFpEF (the ALDO-DHF trial, but only 5% of participants [n=22] had AF).[43]

However no data are available on effects of aldosterone antagonists on diastolic dysfunction and exercise tolerance in AF.

We are aware that the effectiveness of spironolactone was recently tested in two clinical trials of HFpEF. The ALDO-DHF[43, 44] study was essentially a study of HFpEF in hypertensive subjects, which is another major cause of HFpEF as well as AF. Hypertension was present in 92% of participants in the ALDO-DHF study, with AF only present in 5% of the study participants at presentation (n=22). Thus, evidence in symptomatic permanent AF would be sparse, and ALDO-DHF would not address the

issue. In contrast the IMPRESS-AF trial will specifically focused on permanent AF as the study population per se.

The second study (TOPCAT)[45, 46] included a higher proportion of subjects with any concomitant form of AF (typically dominated by paroxysmal AF). The TOPCAT study defined preserved LV function as LVEF <45%, thus leading to recruitment of a proportion of participants with impaired LVEF according to contemporary definitions.[1, 47]

Thus, the effectiveness of spironolactone in the target population of the proposed IMPRESS-AF trial remains unaddressed.

Exercise capacity (primary outcome)

Exercise capacity is a well-established predictor of cardiovascular and overall mortality.[48] Cardiopulmonary exercise testing (CPET) with determination of peak VO_2 is the 'gold-standard' assessment of exercise capacity, that provided unique information on cardiovascular and ventilatory systems' responses, simultaneously, under precise conditions of metabolic stress.[49] This makes CPET ideal non-invasive tool to establish the functional status of the heart, which provides a wealth of clinically relevant diagnostic and prognostic information.[50-52]

AF and Brain Natiuretic Peptide (BNP)

New-onset AF is associated with an elevation of BNP levels, with values that peak within 24–36 hours after AF onset.[53] BNP concentrations gradually decrease in parallel to attainment of heart rate control, although many AF participants remain symptomatic long term.[53, 54] Various factors are implicated in BNP release in AF, including an increase in atrial load, tachycardia, and deterioration in systolic function.

Our pilot data

We used an echocardiographic measure of LV fibrosis (cIB) in order to obtain pilot data on status of LV fibrosis in AF.[31] We found that subjects with both paroxysmal and permanent AF had significantly reduced LV cIB (i.e., more severe fibrosis than control subjects without AF) (Table 1). Low cIB was independently associated with high E/E' ratio (a measure of diastolic dysfunction) after adjustment for age, sex, systolic blood pressure, history of hypertension, diabetes, and CAD (r=-0.24, p=0.032), but it was not significantly associated with LV EF. More severe LV fibrosis (i.e., lower cIB) was significantly correlated with advanced age (r=-0.40, p<0.001), higher systolic blood pressure (r=-0.37, p<0.001), heart rate (r=-0.21, p=0.048), and left atrial volume (r=-0.23, p=0.029).

Table 1.Comparison of LV fibrosis between participants with AF and control subjects

	Permanent AF (n=49)	Paroxysmal AF (n=44)	Disease controls (n=42)	Healthy controls (n=48)	p
cIB, dB	11 (8-18)*†‡	16 (12-19)†‡	22 (19-27)	28 (23-30)	<0.001

*p<0.05 vs. paroxysmal AF, †p<0.05 vs. disease controls, ‡p<0.05 vs. healthy controls. Data median (interquartile range)

The applicants have significant experience of assessing exercise capacity in a variety of settings,[55-57] including research projects in cardiovascular diseases (e.g. AF and HF).[55, 58, 59]

In summary: **Our pilot data demonstrate features of abnormal LV fibrosis in AF and ability of spironolactone to improve diastolic function in participants with preserved LV EF.**

1.2. Trial Rationale

Aims of the trial:

This trial aims to determine whether the aldosterone antagonist, spironolactone improves exercise tolerance and health-related QoL compared to placebo (both added to the current guideline-recommended optimised care) in symptomatic participants with permanent AF.[23]

Original hypotheses:

We hypothesise that in symptomatic participants with permanent AF treatment with spironolactone will improve exercise tolerance as a surrogate for cardiovascular mortality/morbidity *(primary outcome)*, and will improve QoL and diastolic function, as well as reduce the rate of all-cause hospital admissions, and increase rate of spontaneous cardioversion to sinus rhythm *(secondary outcomes)*.

This dose has been shown to improve outcomes in systolic HF, improve diastolic function in HFpEF and to reduce collagen turnover, a marker for fibrotic signalling, in the RALES population.[35] The same dose of the spironolactone within 1 year significantly improved diastolic function in participants with HFpEF from the ALDO-DHF trial.[43]

All participants will receive current optimised treatment following established clinical guidelines on management of AF, HF and hypertension, including anticoagulation, rate control, ACE inhibitors, diuretics, and devices such as defibrillators or cardiac resynchronisation therapy as indicated.[23]

Over the two year trial period, we hypothesis that the greatest clinical benefits in QoL will be observed due to taking Spironolactone over the 2 year duration of the trial. We will also undertake exploratory analysis at 12 months to see if the effect can be detected earlier.

The IMPRESS-AF trial will thereby provide evidence to decide whether spironolactone improves exercise capacity, symptoms, and quality of life in participants with AF.

The results of this trial will thereby allow the clinical use of spironolactone as a new form of therapy for symptomatic participants with AF. Spironolactone is available within the NHS for the management of participants with hypertension and HF with reduced systolic function. The QoL data collected as part of the trial may be used to inform clinical guidelines and health policy.

The applicants are involved in a range of studies addressing cardiovascular morbidity and mortality. These provide a platform for the proposed trial. The trial will be conducted in compliance with this protocol, GCP and the applicable regulatory requirements.

Clinical Implications

The IMPRESS-AF trial will provide evidence on the clinical effectiveness of a readily available treatment in participants with AF. This large population of participants suffers markedly from reduced quality of life and is in clear need for additional therapies to improve their management. The data collected in this controlled trial dataset would achieve high external validity and will inform future guidance on the usefulness of spironolactone in this participant population. If our hypotheses were confirmed in this trial, IMPRESS-AF would provide a basis to roll out spironolactone therapy to prevent deterioration and improve symptoms and exercise capacity in these participants.

Mechanistic aspects of spironolactone-mediated effects

As mentioned in the introduction it is likely that antifibrotic effects of the spironolactone can play a clinically relevant role in the trial population. As previously suggested by the EME Board and in order to reduce costs of the project and to make the trial more straightforward and focused, we have removed the mechanistic parts of the trial (e.g. assessment of cardiac fibrosis using cardiac magnetic resonance imaging, laboratory measures, etc).

2. AIMS, OBJECTIVES AND OUTCOME MEASURES

2.1 Aims and Objectives

The primary objectives of the trial are to determine if treatment with Spironolactone will improve exercise tolerance as a surrogate for cardiovascular mortality and morbidity, in participants with AF. The trial will also assess the effects of spironolactone on Qol and diastolic function, as well as rates of all-cause hospital admissions and spontaneous return to the sinus rthythm.

2.2 Outcome Measures

The **Primary** efficacy end point will be the improvement in exercise tolerance at 2 years. This will be assessed by the difference between trial groups in peak VO_2 on CPET.

The **Secondary** efficacy end point will be the level of improvement in quality of life and diastolic function, and also the improvement the rate of all-cause hospital admissions and spontaneous return the sinus rthythm, with spironolactone. This will be assessed by:

a) improvement in exercise tolerance measured by 6-minute walking test (a simple test of exercise performance) at baseline and at 2 years
b) improvement in quality of life (using the validated Minnesota Living with Heart Failure [MLWHF][60-62] and EuroQol EQ-5D-5L [EQ-5D][63, 64] questionnaires self-completed by participants, which will be completed at each months 12 and 24)
c) improvement in LV diastolic function (E/E' on echocardiography) will be assessed at baseline and at 2 years
d) improvement in rates of all-cause hospitalisations during 2 year follow-up.[62, 63]
e) spontaneous return to sinus rthythm (ECG) after 2 years of treatment.

3. TRIAL DESIGN

3.1 Trial Design

This is a double-blind, 2 year randomised, placebo-controlled single centre trial, recruiting 250 participants from primary and secondary care. Eligible participants will present with symptoms of permanent AF levels and will be randomised to receive a 2 year treatment with either Spironolactone or placebo.

Prior to screening and recruitment of participants, the trial protocol will be finalised, practices recruited, research staff trained, initial eligibility screening in general practices undertaken to identify potential participants (Figure 3). Trial committees will meet to plan their respective committee charters and operating procedures.

Preliminary eligibility for the trial will be checked by the participants' GPs prior to patients being invited for the screening visit to the RC-CCS. Potential participants will be invited to attend an appointment at the trial site for an initial screening visit. They will be asked to bring their medication with them to the visit. At the screening visit, participants will be consented into the study and screened for eligibility. Eligible participants will be given the option of having the baseline visit straight after the screening visit or on a separate date. During the baseline visit, they will be randomised into the trial, subject to meeting the inclusion and exclusion criteria. Baseline parameters will be recorded. Participants will be randomised to receive a 2 year treatment with either Spironolactone or placebo and will be required to attend follow-up visits during the 2 year treatment period. The end of the trial will be defined as the date of the last visit of the last participant undergoing the trial.

After the recruitment the participants will have follow-up visits at 1, 3, 6, 9, 12, 15, 18, 21 months for assessment of clinical status, measurement of blood pressure, potassium, sodium, full blood count/hematocrit and renal function. Trial medication will be dispensed at baseline, 6 months, 12 months and 18 months. Quality of life will be assessed at baseline, 12 months and 24 months using the EQ-5D and the MLWHF questionnaires.

The final visit will be done at 24 months from the randomisation to assess the primary and secondary outcomes. The statistical analysis will be completed in year 4 of the trial (months 40-42).

3.2 Trial Setting

Trial participants will be recruited from primary care AF disease registers (GP practices) as well as outpatient AF clinics (run by Professor Gregory YH Lip & Professor Paulus Kirchhof) in two large teaching hospitals: (i) City Hospital and (ii) Sandwell Hospital (the AF clinics).

This will allow enrolment of a representative population of patients with AF. The list of trial sites will be filed within the IMPRESS-AF Trial Master File (TMF).

4. TRIAL POPULATION

4.1 Identification of participants

A total of 2560 potential patients will be screened within through Primary Healthcare Providers (i.e. GP Practices and Community Pharmacy). Screening will be within GP practices via GP registers. In addition, suitable patients from secondary care outpatient clinics will also be invited.

- Trial participants will be recruited from primary care AF disease registers as well as outpatient clinics in two large teaching hospitals
- PCCRTU will coordinate participant searches, using the clinical research network (CRN). The CRN will facilitate participant searches which will be carried out by staff at the GP practices. Professor Gregory YH Lip & Professor Paulus Kirchhof will identify suitable patients within their AF clinics
- The GPs will screen disease registers for participants with AF (with all of the inclusion and exclusion criteria applied), with the intention to screen 2560 potential participants.
- Potential participants (approximately 1280) who correspond to the trial requirements will be identified and invited to participate by their GP practice. Invitation letters and Patient Information Sheets will be sent to potential participants and will be produced by either a mail merge at the practice or via Docmail, a secure online mailing service.
- People who do not respond to the invitation letter within one calendar month will be sent a single reminder letter and Patient Information Sheet.
- Approximately 640 interested participants will reply to the Research Team at the University of Birmingham with an expression of interest to take part in the trial.

4.2 Eligibility

4.1.1 Inclusion Criteria

- Age 50 years old or over
- Permanent AF as defined by the European Society of Cardiology (ESC) criteria

Inclusion criteria justifications:
To improve generalizability we do not include a requirement for evidence of diastolic dysfunction as the trial patients would have impaired diastolic function due to AF. Patients of both genders will be represented in the trial. Patients aged below 50 years will not be included as they represent a very small proportion (<10%) of patients with AF with HFpEF. This will provide a representative population of the targeted patients avoiding issues of possible pregnancies whilst on the trial medication.

4.1.2 Exclusion Criteria

- Severe systemic illness with life expectancy of less than 2 years from screening
- Left ventricular ejection fraction (LVEF) <50% (echocardiography)
- Severe *chronic obstructive pulmonary disease (COPD*) (e.g., requiring home oxygen or chronic oral steroid therapy)
- Severe mitral/aortal valve stenosis/regurgitation
- Significant renal dysfunction (serum creatinine 220 µmol/L or above), anuria, active renal insufficiency, rapidly progressing or severe impairment of renal function, confirmed or suspected renal insufficiency in diabetic patients/ diabetic nephropathy
- Increase in potassium level to >5mmol/L
- Recent coronary artery bypass graft surgery (within 3 months)
- Use of aldosterone antagonist within 14 days before randomisation
- Use of or potassium sparing diuretic within 14 days before randomisation
- Systolic blood pressure >160 mm Hg

- Addison's disease
- Hypersensitivity to spironolactone or any of the ingredients in the product
- Any participant characteristic that may interfere with adherence to the trial protocol
    - In-ability to understand and complete questionnaires (with or without use of a translator/translated materials).

5. CONSENT

It will be the responsibility of the Investigator to obtain written informed consent for each participant prior to performing any trial related procedure. In the majority of cases the participants will be consented by the clinical research fellow listed on the delegation log. Taking of consent may be delegated to other medically qualified members of the research team.

A Participant Information Sheet (PIS) will be provided to participants. The Investigator or delegate(s) will ensure that they adequately explain the aim, trial treatment, anticipated benefits and potential hazards of taking part in the trial to the participant. They will also stress that participation is voluntary and that the participant is free to refuse to take part and may withdraw from the trial at any time. The participant will be sent the PIS in the post as a part of the invitation, allowing the participant the opportunity to consider their participation in the trial. A screening appointment will be made at which the participant will have the opportunity to ask any questions. If the participant expresses an interest in participating in the trial they will be asked to sign and date the latest version of the Informed Consent Form (ICF). The consent form will ask the patient to provide explicit consent for the regulatory authorities, members of the research team and or representatives of the sponsor to be given direct access to their medical records and this will be reflected in the consent documentation.

The Investigator or delegate will then sign and date the form. A copy of the ICF will be given to the participant, a copy will be filed in the medical notes, and the original placed in the Investigator Site File (ISF). Once the participant is entered into the trial, the participant's trial number will be entered on the Informed Consent Form maintained in the ISF.

Details of the informed consent discussions will be recorded in the participant's medical notes. This will include date of discussion, the name of the trial, summary of discussion, version number of the PIS given to participant and version number of ICF signed and date consent received. Participants will be advised that data from thir QoL questionnaires will not be used directly to inform their clinical care and that if they have any concerns regarding their well-being that the participant should raise this directly with their clinical team. Throughout the trial the participant will have the opportunity to ask questions about the trial. Any new information that may be relevant to the participant's continued participation will be provided. Where new information becomes available which may affect the participants' decision to continue, participants will be re-consented. The participant's right to withdraw from the trial will remain.

Electronic copies of the PIS and ICF will be available from the PC-CRTU trials team and the header will contain details of the local institution. Details of all participants approached about the trial will be recorded on the Participant Screening/Enrolment Log and with the participant's prior consent, their General Practitioner (GP) will also be informed that they are taking part in the trial.

6. ENROLMENT AND RANDOMISATION

6.1 Enrolment

Screening:

- Informed consent taken
- Eligibility criteria will be checked with the participant.
- Brain Natiuretic Peptide (BNP) level test using point of care device
- BP measurement
- If the participant has had blood tests performed at City Hospital within the 30 days preceeding the screening visit, results from these tests will be used for assessment at screening. If the results are abnormal and there is a clinical reason to repeat or no results are available for the patient, blood samples will be taken and the following tests performed: renal function, potassium, sodium, HBA1c (for diabetic patients only), lipid levels and full blood count (including hematocrit)
- Echocardiogram and ECG will be performed

6.2 Randomisation

Patients meeting all eligibility criteria and providing written informed consent will be randomised at the baseline visit. Participants will first be stratified on the basis of their baseline peak VO_2 (two stratification groups; VO_2 ≤16 ml/min/kg, and VO_2 >16 ml/min/kg). A secure web-based randomisation system will be used to allocate a unique investigational medicinal product (IMP) number to each participant.

The randomisation process will use two lists. The first list (the Randomisation List) comprises two stratification groups for patients with peak VO_2 ≤16ml/min/kg and peak VO_2 >16ml/min/kg, with a total number of 256 rows per stratification group (to allow for the hypothetical possibility that all trial participants could fall into the same stratification group). Each row will be marked as 'A' or 'B', referring to treatment A or treatment B respectively. A block randomisation approach will be taken, whereby each stratification group is divided into blocks of four rows, with both A and B occurring twice within every block of four (i.e. AABB, ABAB, ABBA, BBAA, BAAB and BABA). This will ensure that the number of A's or B's can only differ by a maximum of two within any sequence of randomisation within each stratification group (the number of A's and B's will usually be the same or one apart). The randomisation process will work through the relevant stratum of the Randomisation List in a sequential order, to assign each patient to treatment A or B. The second list (the IMP Number List) consists of the unique numbers 001P to 250P in a random order, and assigned randomly to A or B, but in a 50:50 ratio. Upon determination of A or B from the Randomisation List, the computer will select a unique number from the corresponding group in the IMP Number List. This number will be assigned to the newly randomised patient, who will then be supplied with the medication bottle with the matching number.

The online randomisation described above will be incorporated into a secure bespoke trial database; however contingency has been planned for paper-based randomisation in the unlikely event of a temporary system failure. A telephone back up will be available via the PC-CRTU randomisation line, which will be staffed Monday to Friday during office opening hours.

EMERGENCY RANDOMISATION PC-CRTU

During office hours only for use during online system failure

☏ **0800 694 6943**

🖷 **0121 414 3050**

6.3 BLINDING

Trial participants, the trial team in contact with the patient, care providers, outcome assessors and data analysts will all remain blinded to the treatment after assignment to interventions.

Blinding of the trial drug identity will take place at the time of packaging and labelling. The trial drug will be overencapsulated and packaged by Catalent Pharma Solutions, who will be supplied with the IMP Number List by the PC-CRTU computer programmer. The IMP Number List will also state which of A or B represents the spironolactone, and which the placebo, and Catalent Pharma Solutions will use this information to label the medication bottles accordingly. Overencapsulation and bottle labelling will be performed in one run for spironolactone and a completely separate run for placebo. Medication bottles will be labelled only with the unique IMP number, and the label will not indicate whether the contents are active or placebo. Catalent Pharma Solutions will also produce six spare bottles of trial medication (three each of spironolactone and placebo, randomly assigned by the computer programmer to IMP Numbers 251P-256P). In the event of a patient losing their medication, the computer will allocate to that patient another number corresponding to a spare medication bottle for the same trial arm.

Only the computer programmer and Catalent Pharma solutions will see the IMP Number List (and thus be unblinded to the trial arms), however 256 individually numbered security-sealed envelopes, each containing the respective codebreak, will also be supplied to the Pharmacy Department at City Hospital (who are independent of the trial, and operate 24 hours a day). City Hospital Pharmacy Department will be responsible for unblinding in the event of an emergency codebreak situation occurring. Codebreaks of the trial drug will be restricted to emergency situations only; these will be rare, as any sick patient will be treated symptomatically, and their management will not usually be altered by knowing the identity of the trial compound (i.e. spironolactone or placebo). Unblinding should be avoided unless knowledge of the trial treatment is essential for the correct clinical care of the patient, e.g. in the cases of acute liver injury, acute renal failure, Stevens-Johnson syndrome or agranulocytosis, if suspected to have been caused by spironolactone. Cases that are considered serious, unexpected and possibly, probably or definitely related will be unblinded.

In the event of a codebreak situation occurring, City Hospital Pharmacy Department will be alerted and will follow the instructions on the codebreak form; all fields must be completed before performing the codebreak. The PC-CRTU should be informed as soon as possible if the code has been broken. This must be done by sending a copy of the completed codebreak form to the PC-CRTU via email or fax. The codebreak envelope must also be resealed as soon as possible; the time and date of the codebreak should be documented, along with the name of the person performing the codebreak, and the reason for the codebreak.

Once the code is broken, the patient will be withdrawn from the trial treatment, as they will become unblinded to their trial drug. However, where applicable, these patients will be asked to complete the QoL questionnaires at the 12 month and 24 month visits. Please refer to the patient withdrawal section for more information.

6.4 MECHANISTIC SUBSTUDIES

The blood samples collected during the study visits may be used or stored for future use within the study centre at City Hospital for other ethically approved research projects if relevant consent provided by the patient (Appendix 1).

7. TRIAL TREATMENT / INTERVENTION

7.1 Treatment Supply and Storage

7.1.1 Treatment Supplies

The treatment will be manufactured by Catalent Pharma Solutions and will be shipped directly to the University of Birmingham Centre for Cardiovascular Sciences, City Hospital, Birmingham, B18 7QH. The drug will be produced and delivered in a single batch (as per submitted contract) but if required, this can be negotiated to be produced and delivered in separate batches. Oversight of the drug shipping and handling will be coordinated and managed by the University of Birmingham Centre for Cardiovascular Sciences, City Hospital, Birmingham, B18 7QH.

7.1.2 Packaging and Labelling

Catalent Pharma Solutions will also be responsible for the packaging and labelling of the treatment. Placebo and spironolactone will be identical in appearance and packaging and manufactured as 25-mg tablets in accordance with national and international standards.

Sample label:

Subject code: XXXX

Batch Number: XXXXXXXXXX

Expiry Date: DD/MM/YYYY

Quantity of capsules in each container: XXXX

For clinical trial use only

Patient name: ______________

Date of Supply: ______________

Directions: one capsule per day or as directed

Product: Spironolactone 25mg or placebo

Storage conditions: Store in a dry, dark place below 25°C

Keep out of the reach of children

For use in IMPRESS-AF trial (EudraCT number: 2014-003702-33)

Investigator: Prof. G. Lip; Sponsor: University of Birmingham

Study Telephone number for all enquiries: 0121 507 6657

L1400467

7.1.3 Drug Storage

The drug will be stored in a dedicated storage facility within the University of Birmingham Centre for Cardiovascular Sciences Clinical Trials Unit. The main hospital pharmacy will have oversight. The drug will be stored in a secured room, which meets storage requirements and is compliant with GCP regulations. Checks will be performed, in line with GCP guidelines, including temperature control logs and an SOP detailing temperature excursions, annual audits from pharmacy, secure storage area and limited access.

Any remaining drug at the end of the trial, or returned drug from patients who have withdrawn, will be disposed of by the Centre for Cardiovascular Sciences in conjunction with the NHS pharmacy. The Pharmacy waste disposal will be done using sealed containers prior to incineration off site (Company SITA)

7.1.4 Dispensing of treatment

The Centre for Cardiovascular Sciences with be responsible for dispensing the trial medication to the participant on receipt of a prescription. The hospital NHS pharmacy will have oversight and will perform an annual audit. The drug will be labelled by Catalent Pharma Solutions before being shipped to the RC-CCS.

7.2 Treatment and Dosing Schedule

Trial participants will receive either Spironolactone or matched placebo according to the following instructions:

- a single 25 milligram tablet
- to be taken orally once per day (typically to be taken during the morning, but can be taken regularly at other time of day if preferable).
- to be taken with a drink to help swallowing of the drug
- for a 24 month period

In the case of an increase in potassium level to 5.1-5.5 mmol/L or in the presence of other non-life-threatening side effects (such as gynaecomastia), the trial drug will be down-titrated to 25 mg each second day. In such cases, the investigators are advised to re-up-titrate the trial medication if the reason for down-titration has resolved. Routine laboratory surveillance of serum potassium, sodium, full blood count with hematocrit, and renal function will be done by protocol at each visit and within 1 week of any dose adjustment.

Drug toxicity will be defined as an increase in potassium level to >5.5 mmol/L. In the case of toxicity or suspected toxicity, the trial medication will be stopped for the duration of the trial, but the patient will be requested to attend the remaining follow up visits.

7.3 Drug Interaction or Contraindications

For the purposes of analysis, we will record if the participant is taking any potassium supplements.

7.4 Accountability Procedures

The participants will be given bottles with the trial drug sufficient for 6 months of treatment. A pill count will be performed by the nurse at each visit. The remaining number of pills in the pack will be checked and recorded in the patient CRF for that visit. At the visits at 6 months, 12 months and 18 months, the previous bottle and any remaining pills will be returned and the next supply will be dispensed. Bottles will be returned to the nurse at the clinic visit and will be stored in the RC-CSS pharmacy until the end of the trial.

7.5 Treatment Modification

Participants will be instructed to take the trial medication at 25 mg once daily after randomisation. There is no up-titration planned. Temporary down-titration is allowed in the presence of reversible, non-life-threatening side effects. Please refer to the treatment and dosing schedule for further information.

7.6 Concomitant medications

Blood pressure will be controlled during the duration of the study with particular attention to blood pressure levels after beginning of the study drug and after any changes in antihypertensive agents and their doses.

Electrolyte levels (i.e., potassium and sodium) will be monitored in all study patients to avoid their abnormalities, particularly in participants receiving anti-diabetic treatments, ACE inhibitors and Angiotensin-II receptor antagonists.

Tacrolimus potassium supplements, potassium-sparing diuretics will not be used in the study patients.

Administration of NSAIDs (especially indometacin and mefenamic acid) and ciclosporin will be avoided in the study patients unless clinically indicated.

In patients receiving lithium, monitoring of lithium levels will be arranged.

As all study patients will receive warfarin or dabigatran etexilate or factor Xa inhibitors. In patients receiving warfarin INR will be controlled.

8. TRIAL PROCEDURES AND ASSESSMENTS

			Follow-up								
Visit	Screening	Baseline	Month 1	Month 3	Month 6	Month 9	Month 12	Month 15	Month 18	Month 21	Month 24
		Additional visits will be arranged to reassess potassium levels if patient's blood results show a potassium level of >5.0 mmol/L									
Eligibility Check	X	X									
Informed consent	X										
Relevant medical history taken	X										
Concomitant medication	X	X	X	X	X	X	X	X	X	X	X
Standard clinical examination including BP check	X	X	X	X	X	X	X	X	X	X	X
Clinical Biochemistry											
Full blood count (FBC)	X		X	X	X	X	X	X	X	X	X
Renal function, potassium, sodium	X		X	X	X	X	X	X	X	X	X
HBA1c (for diabetics)	X										
Lipid levels	X										
Electrocardiogram (ECG)	X										X
Echocardiogram	X										X
Brain Natriuretic Peptide test (BNP)	X										X
Randomisation		X									
Dispensing of IMP		X			X		X		X		
Cardiopulmonary exercise testing (CPET)		X									X
6 minute walk test		X									X
Quality of life questionnaires		X					X				X

Figure 1 - a table of assessment detailing the timeline of trial procedures alongside the assessments that will be carried out at each stage.

8.1 Summary of assessments

Trial participants will be recruited from primary care AF disease registers within the 64 GP practices as well as outparticipant clinics in two large teaching hospitals:

(i) City Hospital
(ii) Sandwell Hospital [the AF clinics run by Professor Gregory YH Lip & Professor Paulus Kirchhof].

This will allow enrolment of a representative population of participants with AF.

- PCCRTU will coordinate participant searches, using the Clinical Research Network: Primary Care (CRN:PC). The CRN:PC will facilitate participant searches which will be carried out by staff at the GP practices.
- The GP's will screen disease registers for participants with AF (with all of the inclusion and exclusion criteria applied), with the intention to screen 2560 potential participants.
- Potential participants (approximately 1280) who correspond to the trial requirements will be identified and invited to participate by their GP practice. Invitation letters and Patient Information Sheets will be sent to potential participants and will be produced by either a mail merge at the practice or via Docmail, a secure online mailing service.
- People who do not respond to the invitation letter within one calendar month will be sent a single reminder letter and Patient Information Sheet.
- Approximately 640 interested participants will reply to the Research Clinic at the University of Birmingham Centre for Cardiovascular Sciences, Sandwell and West Birmingham Hospital (RC-CCS) with an expression of interest to take part in the trial. RC-CCS will keep a log of all replies and provide updates on figures to the PC-CRTU as agreed.
- The RC-CCS will invite interested participants for a screening visit at the RC-CCS. The inclusion and exclusion criteria will be checked and Informed consent will be received. The following tests will be performed: BNP, echocardiogram, standard clinical examination (including BP measurement), ECG and a blood sample taken to test renal function, potassium, sodium, HBA1c (for diabetic participants only), lipid levels and full blood count (including hematocrit).
- Participants who meet the inclusion criteria will be invited to a baseline appointment and randomisation. They will be given the option of having the baseline appointment straight after the screening visit or at a later date. At the baseline appointment, eligibility, medical history and medication will be reviewed. Participants will be asked to bring their medication to every visit and told that, for participant safety, the appointment will not be able to go ahead without it. The participant will undergo standard clinical examination (including BP measurement), CPET and a 6 minute walking test. Quality of life questionnaires will be also completed (prior to randomisation). Participants' GPs will be informed of their participation in the trial.
- Double blinded block randomisation will be used to allocate participants to either a 2-year treatment with spironolactone or placebo, and trial medication will be dispensed.
- Following recruitment, all participants will be seen for follow-up visits at 1 month of randomisation and then quarterly during the 2 year follow-up period. All participants need to be seen within +/-1 week for month 1 visit and +/-2 weeks for all other follow up visits.Standard clinical examination (including BP measurement), concomitant medication review, blood samples will be taken at each of these follow-up visits to test renal function, potassium, sodium and full blood count (including hematocrit). IMP will be dispensed at months 6, 12 and 18 and QoL questionnaires will be completed at month 12.
- The participants will attend a final visit where the following will be done: standard clinical examination (including BP measurement), concomitent medication review, blood samples to test renal function, potassium, sodium and full blood count (including hematocrit),

echocardiogram, ECG, BNP, CPET, 6 minute walk test. The QoL questionnaires will be completed.

- During the trial, should a test not be able to be performed, for example difficulty obtaining a blood sample or technical issues with equipment, participants will be asked if the visit can be rebooked.
- Participants' GPs will be informed of any abnormal results during the trial.

Work done	Months of the project													
	1-3	4-6	7-9	10-12	13-15	16-18	19-21	22-24	24-27	28-30	31-33	34-36	37-39	40-42
Protocol refined, practices recruited, research staff trained, initial eligibility screening in general practices undertaken to identify potential participants														
Screening and recruitment of the participants*														
Two-year follow-up of the trial participants**														
Data analysis														

*See left side of the figure 2 for more details; **See right side of the figure 2 for more details

Figure 3. Gantt chart for the IMPRESS-AF trial.

8.2 Trial Procedures

Establishment of permanent AF

Permanent AF will be defined in accordance with the ESC criteria as a cardiac arrhythmia with the following characteristics: (1) The surface ECG shows 'absolutely' irregular RR intervals; (2) There are no distinct P waves on the surface ECG; (3) The atrial cycle length (when visible), is usually variable and <300 bpm; (4) duration of the AF >12 months with no plans in place for pharmacological or electrical cardioversion (rate control approach)

Cardio-pulmonary exercise testing (CPET) and 6-minute walk test

CPET testing with oximetry will be performed as described previously to assess peak oxygen uptake (peak VO_2) and to define cardiovascular etiology of the symptoms.[65-67] Recently, CPET has been shown to be a highly accurate and reproducible measure of exercise tolerance in participants with preserved LV contractility.[68] Exercise testing will be performed with participants in the upright position on an electronically braked bicycle, with expired gas analysis under continuous electrocardiographic and blood pressure monitoring.[69] Participants will be encouraged to exercise to exhaustion. Peak VO_2 values will be averaged from the final 30 seconds of the exercise test. Additionally, ventilator anaerobic threshold will be evaluated by standardized methods using ventilator equivalents.[69]

The Statement of the American Thoracic Society and American College of Chest Physicians recommends that an increased VE/MVV ratio (e.g., > 85%) occurring at a relatively low work rate (e.g., 50 W) strongly suggests that ventilator factors are contributing to exercise limitation.[70]

The CPET test will be performed using the L COSMED CPET system based on cycle ergometer procedural instructions. CPET testing will be performed as per the RC-CSS SOP. Interpretation of the VO_2 maximum value result will be used will be used as a measure of exercise tolerance (i.e. higher value means better exercise tolerance). The research fellow will be recording these results and there is no risk of these results indicating the treatment that the patient is receiving. The device will be calibrated as part of an annual service.

Exercise Tolerance Test – 6 minute walk test

In addition to CPET the participants will be asked to undertake a 6-minute walk test as a simple measure of exercise tolerance.[71] The test will be performed as recommended previously[72] and will help to estimate its utility for the participant selection, should the trial result need to be implemented into the clinical practice.

No specialist equipment is required for this procedure and instructions on how the test should be performed will be detailed within the Work Instruction and will be in accordance to the trial protocol. For the purpose of the trial only the distance the patient can walk needs to be recorded for analysis. The research fellow will be recording the results as these results are not indicative of the treatment that the patient is receiving.

Echocardiography

M-mode, 2D, Doppler and TDI transthoracic echocardiography will be performed using Phillips iE33 ultrasound system (Bothel, WA, USA). Modern off-line QLAB software [Xcelera, Phillips iE33 Ultrasound Quantification Module, USA] will be used for quantification of LV systolic and diastolic function.[73] Single beat E/E' (early mitral inflow velocity/TDI derived early septal mitral annular diastolic velocity) will be used to assess diastolic function in AF.[74, 75] The parameter was strongly correlated with LV diastolic filling pressure in AF ($r=0.79$, $p<0.001$) and it is relatively independent of left atrial pressure.[74] An average values from 10 consecutive cardiac cycles will be calculated

ECGs will be performed using the Marquette Hellige – model Mac 1200. ECGs will be performed as per the RC-CSS SOP. The ECG will be interpreted by a physician (clinical research fellow) and the results will not compromise blinding. The device will be calibrated yearly and yearly calibration certificates will be made available by the RC-CSS.

Brain Natiuretic Peptide (BNP) level test using point of care device

These near patient tests will be performed at a number of patient appointments. They will be performed using a commercial BNP test system (Alere Triage® BNP Test). The BNP machines will be used strictly according to the manufacturer's instructions. The device will be calibrated yearly and certified. The results of this test will not be indicative of the treatment that the patient is receiving.

Quality of life assessment

The QoL of patients enrolled in IMPRESS-AF will be assessed using generic (EQ-5D)[76] and disease-specific (MLWHF)[62] measures. The EQ-5D will allow comparison with a general population and to provide utility estimates to inform future clinical effect analyses. The MLWHF will be used to provide more detailed information on changes in the patient HF related QoL to inform clinical care and guidelines.

The EQ-5D is a self-administered, validated, generic preference-based measure of health status that comprises a 5-question multi-attribute questionnaire and a visual analogue self-rating scale.[77] Respondents are asked to rate severity of their current problems - 5 levels: no problems, slight problems, moderate problems, severe problems, and extreme problems. The respondent is asked to indicate his/her health state by ticking (or placing a cross) in the box against the most appropriate statement for five dimensions of health: mobility, self-care, usual activities, pain/discomfort, and anxiety/depression. Patients can therefore be classified into 3125 health states. EQ-5D health states may be converted into an EQ-5D index score ranging from 0.594 to 1.0 (where 1 is full health and 0 is dead) using a set of weighted preferences produced from the UK population.[78-80] The EQVAS is a visual analogue scale on which respondents are asked to rate their own health state relative to full health (score=100) or worst imaginable health state (score=0). This questionnaire will help to assess overall QoL of the study participants.

The MLWHF is a validated, disease-specific, self-administered questionnaire.[62] This instrument consists of 21 questions focussing on the impact of heart failure on QoL. Patients are asked to rate the extent to which their HF has prevented them from living as they wanted during the last month using questions rated on a scale of 0 (no effect) to 5 (very much). The questionnaire is scored by summating the responses to all 21 questions; thus resulting in a score from 0 to 105 with a higher score reflecting poorer QoL. This questionnaire will help to assees QoL specifically related to HF symptoms which is of particuly importance for the targeted trial population and expected benefits.

Blood Pressure Measurement
Blood pressure readings will be taken using the Omron 705IT (HEM-759P-EZ) blood pressure machine. A SOP will be made available which will details procedural instructions. Systolic and diastolic blood pressure will be recorded and interpreted in accordance with NICE guidelines on Hypertension (CG127). The device will be calibrated yearly and yearly calibration certificates will be available. The results are not indicative of the treatment that the patient is receiving and will therefore be recorded by the research fellow.

Blood test analysis
All clinical laboratory procedures will be undertaken by:

Pathology Department, City Hospital, Dudley Road, Birmingham, B18 7QH.
Tel. 0121 507 3130

Blood tests will be taken after patient written informed consent has been received and electronic requisitions will be completed. Specimens will be labelled as per Trust guidelines, a copy of the requisition will be retained.

Blood test results can be reviewed electronically, a copy will be printed. These will reviewed and signed on a daily basis by the trials physician listed on the delegation log with this delegated duty

Blood samples will be used for routine laboratory checks done in City Hospital laboratory. With the participants consent, part of the blood samples will also be stored frozen in the study centre in City Hospital for future analysis of markers, which would help to better understand the study findings and mechanisms of the disease. Blood samples will not be used to perform any genetic analysis for this study.

For the screening visit, if blood test results are available from City Hospital Pathology Department which were obtained within 30 days of the screening visit, these test results will be used for

assessment at screening. If the results are abnormal and there is a clinical reason to repeat the tests or no results are available, blood samples will be taken.

8.3 Schedule of Assessments

The schedule of assessments and investigations required is described below.

Screening: As described earlier

Baseline (participants who meet the inclusion criteria)

- Eligibility reviewed
- Medical history and medication review
- Standard clinical examination (including BP measurement)
- Cardiopulmonary exercise testing (CPET) with determination of peak VO_2
- 6 minute walking test
- EQ-5D and MLWHF quality of life questionnaires will be completed
- Randomisation to spironolactone or placebo
- Prescription and dispensing of blinded medication by qualified medical research personnel

Follow-up (months 1, 3, 6, 9, 12, 15, 18 and 21)

- Standard clinical examination (including BP measurement),
- Concomitant medication review
- Blood samples to test renal function, potassium, sodium and full blood count (including hematocrit).
- EQ-5D and MLWHF quality of life questionnaires will be completed at months 12 only.
- Information on outcomes such as hospitalisations and major adverse clinical events (MACE) will be collected, as well as safety outcomes (assessment of symptoms and possible side effects)
- Presecription and dispensing of blinded medication by qualified medical research personnel

Final Visit (24 months from randomisation)

- Standard clinical examination (including BP measurement),
- Medication review
- Repeat ECG
- Repeat echocardiogram
- Repeat Brain Natiuretic Peptide (BNP) level test using point of care device
- Repeat Cardiopulmonary exercise testing (CPET) with determination of peak VO_2
- Repeat 6-minute walk test
- Blood sample taken: potassium, sodium, full blood count (including hematocrit) and renal function
- EQ-5D and MLWHF quality of life questionnaires will be completed
  If the patient has an indication for spironolactone, the participants GP will be informed. No further follow up will be arranged. The GP will be informed about the trial completion.

Additional Visits (special conditions)

- Within 1 week of every dose adjustment a blood test will be taken for analysis renal function, potassium and sodium
- The results will be sent to the RC-CCS and reviewed by the trial Research fellow. A fraction of patients may have increase in potassium related to spironolactone. Based on the test results it might be necessary to stop the drug or reduce its dose.
- Participants may voluntarily request withdrawal from treatment. These participants will be requested to return any remaining medication to the RC-CCS. Where patients are only withdrawing from treatment but still wish to take part in the trial, patients will be requested to complete the QoL questionnaires at months 12 and 24.

8.4 Participant Withdrawal

Participants will be withdrawn from the **trial** if written informed consent is revoked by the participant.

Participants will be withdrawn from the **trial medication** in the following cases:

- Potassium level to >5.5 mmol/L.
- Severe renal impairment (e.g. acute renal failure, creatinine >220 μmol/L, creatinine clearance <30 mL/min)
- Significant breast pain or gynaecomastia despite reduction in the trial medication dose (see above)
- Allergy to the trial drug

The participant will be requested to return any remaining medication to the RC-CCS.

Where participants are withdrawing from the trial medication only, but still wish to take part in the trial, they will be asked to complete the QoL questionnaires at months 12 and 24.

9. ADVERSE EVENT REPORTING

9.1 Reporting Requirements

The collection and reporting of Adverse Events (AEs) will be in accordance with the Medicines for Human Use Clinical Trials Regulations 2004 and its subsequent amendments. The CI or PI will assess the seriousness and causality of all AEs experienced by the participant, and the CI will assess the expectedness of all SARs. This will be documented in the source data with reference to the safety information contained within the Summary of Medicinal Product Characteristics (SmPC).

Spironolactone has a half-life of about 1–2 hours, but it has active metabolites with longer half-lives. Therefore the end point for the reporting of serious adverse event will be 7 days after the last dose.

9.2 Adverse Events (AE)

All medical occurrences which meet the definition of an AE should be recorded on the AE pages of the CRF. Please note this includes abnormal laboratory findings. All AEs recorded on the AE CRF must be followed up to resolution and patients must be asked if they have experienced any AEs at every visit.

9.3 Serious Adverse Events (SAE)

Investigators will report all AEs that meet the definition of serious to the PC-CRTU. A SAE Form should be completed for each event and must be faxed to the PC-CRTU trials team immediately.

9.3.1 Events that do not require reporting on a Serious Adverse Event Form

The following are regarded as expected events for the purpose of the trial and should not be reported on an SAE form. These events should be collected on the routine CRFs instead.

(i) development of hyperkalaemia (>5.0 mmol/L)
(ii) a rise in creatinine to >220 µmol/L
(iii) development of gynecomastia
(iv) development of side effects requiring withdrawal of the trial treatment

9.3.2 Monitoring pregnancies for potential Serious Adverse Events

The trial will exclude patients younger 50 years to avoid cases of pregnancies of the female participants. In the event that a male participant's partner becomes pregnant, there is no identified risk of congenital anomalies or birth defects in the offspring. There will therefore be no monitoring of pregnancies outcome.

9.4 Reporting period

Details of all AEs will be documented and reported from the date of consent (screening) until 7 days after the last dose.

9.5 Reporting Procedure – At Site

9.5.1 Adverse Events

AEs should be collected on an AE Form (and where applicable on an SAE Form). An AE Form should be completed at each visit where applicable.

9.5.2 Serious Adverse Events

AEs defined as serious and which require reporting as an SAE should be reported on an SAE Form. When completing the form, the Investigator will be asked to define the causality and severity. The CI will also assess all SARs for expectedness. If the event meets the definition of a SAR that is unexpected (i.e. is not defined in the Reference Safety Information (RSI) it will be classified as a Suspected Unexpected Serious Adverse Reaction (SUSAR).

On becoming aware that a participant has experienced an SAE, the Investigator (or delegate) must complete, date and sign an SAE Form. The form should be faxed to the PC-CRTU trials team using one of the numbers listed below as soon as possible and no later than 24 hours after first becoming aware of the event:

To report an SAE, fax the SAE Form to: *0121 414 3050*

On receipt the PC-CRTU trials team will allocate each SAE a unique reference number which will be forwarded to the site as proof of receipt. If confirmation of receipt is not received within 1 working day please contact the PC-CRTU trials team. The SAE reference number should be quoted on all correspondence and follow-up reports regarding the SAE and filed with the actual SAE in the Site File.

For SAE Forms completed by someone other than the Investigator the Investigator will be required to countersign the original SAE Form to confirm agreement with the causality and severity assessments. The form should then be returned to the PC-CRTU trials team and a copy kept in the Site File.

Investigators should also report SAEs to their own Trust in accordance with local practice.

9.5.3 Provision of follow-up information

Participants should be followed up until resolution or stabilisation of the event. Follow-up information should ideally be provided on a new SAE Form.

9.6 Reporting Procedure – PC-CRTU

On receipt the PC-CRTU trials team will allocate each SAE a unique reference number which will be forwarded to the site as proof of receipt within 1 working day. The SAE reference number will be quoted on all correspondence and follow-up reports regarding the SAE and filed with the actual SAE in the TMF. If the event meets the definition of a SAR that is unexpected (i.e. is not defined in the Reference Safety Information (RSI) it will be classified as a Suspected Unexpected Serious Adverse Reaction (SUSAR).

9.6.1 Provision of follow-up information

Participants should be followed up until resolution or stabilisation of the event. Follow-up information should ideally be provided on a new SAE Form.

9.7 Reporting to the Competent Authority and main Research Ethics Committee

9.7.1 Suspected Unexpected Serious Adverse Reactions

PC-CRTU will report a minimal data set of all individual events categorised as a fatal or life threatening SUSAR to the Medicines and Healthcare products Regulatory Agency (MHRA), main Research Ethics Committee (REC) and RGT within 7 days. Detailed follow-up information will be provided within an additional 8 days.

All other events categorised as SUSARs will be reported within 15 days.

9.7.2 Serious Adverse Reactions

PC-CRTU will report details of all SAEs and SARs (including SUSARs) to the MHRA, main REC and RGT annually from the date of the Clinical Trial Authorisation, in the form of a Development Safety Update Report (DSUR).

9.7.3 Adverse Events

Details of all AEs will be reported to the MHRA on request.

9.7.4 Other safety issues identified during the course of the trial

The MHRA, main REC and RGT will be notified immediately if a significant safety issue is identified during the course of the trial.

Details of all SUSARs and any other safety issue which arises during the course of the trial will be reported to Principal Investigators. A copy of any such correspondence should be filed in the Site File.

9.8 Investigators

**Chief-investigator**	
Professor Gregory YH Lip	*Consultant Cardiologist & Professor of Cardiovascular Medicine,*<br>*Director - Haemostasis Thrombosis & Vascular Biology Unit*

**Co-investigator (s)**	
Professor Paulus Kirchhof	*Chair in Cardiovascular Medicine*
Dr Eduard Shantsila (PI)	*Clinical Postdoctoral Research Fellow in Cardiology*
Dr Paramjit S Gill	*Reader in Primary Care Research*
Dr Melanie Calvert	*Professor of Outcomes Methodology*
Dr James Fisher	*Senior Lecturer in Exercise Physiology*
Dr Sayeed Haque	*Medical Statistician*
Dr Mustafa Abul	Patient representative

9.9 Data Monitoring and Ethics Committee

Data analyses will be supplied in confidence to an independent Data Monitoring and Ethics Committee (DMEC), which will be asked to give advice on whether the accumulated data from the trial, together with the results from other relevant research, justifies the continuing recruitment of further participants. The DMC will operate in accordance with a trial specific charter based upon the template created by the Damocles Group. The DMC will meet every 6 months unless there is a specific reason (e.g. safety phase) to amend the schedule. The DMEC will review all SAEs.

Additional meetings may be called if recruitment is much faster than anticipated and the DMC may, at their discretion, request to meet more frequently or continue to meet following completion of recruitment. An emergency meeting may also be convened if a safety issue is identified. The DMC will report directly to the Trial Steering Committee who will convey the findings of the DMC to the sponsor. The DMEC may consider recommending the discontinuation of the trial if the recruitment rate or data quality are unacceptable or if any issues are identified which may compromise participant safety. The trial would also stop early if the interim analyses showed differences between treatments that were deemed to be convincing to the clinical community.

The trial may be stopped due to poor recruitment.

The DMEC will be responsible for the regular monitoring of trial data. The DMEC will assess the progress of the trial and give advice on whether the accumulated data from the trial, together with the results from other relevant trials, justifies the continuing recruitment of further participants. The committee will meet in person or by teleconference prior to the trial commencing and then three and six months after initiation of the trial. The DMC will make confidential recommendations to the TSC as the decision –making committee for the trial.

Currently agreed DMEC members

**DMEC members**	
Statistician: Dr Paul Ewings, Director of NIHR Research Design Service South West	Address: RDS South West Research Office The Old Building Musgrove Park<br>Hospital Taunton Somerset, TA1 5DA<br>Email: paul.ewings@tst.nhs.uk; Tel.: 01823 342796
Clinician: Dr Derick Todd, Consultant Cardiologist & Electrophysiologist	Address: Clinical Lead for Electrophysiology<br>Address: Liverpool Heart & Chest Hospital, Thomas Dr, Liverpool, L14 3PE<br>Email: derick.todd@lhch.nhs.uk; Tel: Tel. 441516001394
Expert (also clinician): Dr. Tariq Iqbal, Consultant Gastroenterologist & Honorary Reader in Cancer Studies	Address: Queen Elizabeth Hospital and University Of Birmingham, Mindelsohn Way Edgbaston, Birmingham B15 2WB<br>Email: iqbalth@adf.bham.ac.uk; 0121 371 5909, Extension: 15909; Fax: 0121 460 5842

10. DATA HANDLING AND RECORD KEEPING

10.1 Source Data

Source data will be maintained at site and kept in accordance with GCP. They will be made available for monitoring, audit, and inspection. Study specific information will be primarily recorded on the CRF. These items will be clearly identified in the source data agreement.

10.2 CRF and QoL questionnaire Completion

In all cases it remains the responsibility of the Investigator to ensure that the CRF has been completed correctly and that the data are accurate. This will be evidenced by the signature of the investigator (CI or PI) on the CRF. The completed originals will be stored at the PC-CRTU and a copy filed in the Trial Master File.

QoL questionnaires will be administered and completed by the participant at the clinic visit. QoL questionnaire completion and training will be overseen by a named individual who can answer any questions the participant may have regarding the rationale and method of assessment. The participant will be asked to complete the questionnaire during the clinic visit (prior to other clinical assessments). Ideally the questionnaire should be completed by the participant alone (without assistance from friends, family or the clinical or research team). Any assistance or proxy completion will be recorded and flagged to the trials office. On completion, the QoL questionnaires will be checked on site by a member of the research team for missing data. The participant will be given the opportunity to complete any missing data. If for any reason, the participant is unable to complete the QoL questionnaires during the clinic appointment, the QoL questionnaire can be given to the participant to complete at home. The completed QoL should be completed and sent back to the trials office using a supplied stamped addressed study envelope. Any questionnaires returned in the post will be checked for missing data. Participants will be contacted by telephone and given the opportunity to complete any questions they have missed out. QoL questionnaires must be checked and any missing data completed within 1 week of questionnaire being given to the patient.

Staff delegated to complete CRFs will be trained to adhere to the following CRF completion guidelines:

- Entries on the CRF will be made in ballpoint pen, using blue or black ink.
- Errors will be crossed out with a single stroke and the correction will be inserted and the change initialled and dated. If it is not clear why a change has been made, an explanation should be written next to the change. Correction fluid should not be used.
- QoL questionnaires to be completed in accordance with completion instructions.
- Participants will be encouraged to answer all questions when completing the CRFs and the QoL questionnaires.
- QoL questionnaires will be checked for missing data and where feasible participants will be given the opportunity to complete any missing data.
- Data reported on each CRF should be consistent with the source data or the discrepancies should be explained.
- Reasons for missing data should be documented on the CRF / questionnaire.
- Where information is not known or refused, this will be clearly indicated on the form

10.3 Data Management

The IMPRESS-AF database system will be located within PC-CRTU.

The security of the System is governed by the policies of the University of Birmingham. The University's Data Protection Policy and the Conditions of Use of Computing and Network Facilities set out the security arrangements under which sensitive data should be processed and stored. All studies

at the University of Birmingham have to be registered with the Data Protection officer and data held in accordance with the data protection act. The University will designate a Data Protection Officer upon registration of the study. The Study Centre has arrangements in place for the secure storage and processing of the study data which comply with the University of Birmingham policies.

The System shall incorporate the following security countermeasures:

- Physical security measures: including restricted access to the building, supervised onsite repairs and storages of back-up tapes/disks are stored in a fire-proof safe.
- Logical measures for access control and privilege management: including restricted
- accessibility, access controlled servers, separate storage of non-identifiable data etc.
- Network security measures: including site firewalls, antivirus software, separate secure network protected hosting etc.
- System Management: the System shall be developed by the PC-CRTU Programming Team and will be implemented and maintained by the PC-CRTU Programming Team.
- System Design: the system shall comprise of a database and a data entry application with firewalls, restricted access, encryption and role based security controls.
- Operational Processes: the data will be processed and stored within the Study Centre (University of Birmingham).
- Data processing: Statisticians will only have access to anonymised data.
- System Audit: The System shall benefit from the following internal/external audit arrangements:
    - Internal audit of the system
    - An annual IT risk assessment
- Data Protection Registration: The University of Birmingham has Data Protection Registration to cover the purposes of analysis and for the classes of data requested. The University's Data Protection Registration number is Z6195856.

Storage of personal data on manual files; paper - copies of questionnaires, paper copies of results, spreadsheets, lab reports, consent forms and CRFs will be held in secure data storage such as lockable filing cabinets in a restricted access environment. Storage of data on university computer and NHS desktop computers; will be kept in password protected, access limited electronic format. The clinical trial information including CRF will be entered onto an online database and will be anonymised at point of entry. Data entered will be stored on secure servers held at the University of Birmingham.

Manual files containing personal information and allocated identifiers will be kept in a separate location at the RC-CSS to the anonymised data. All computer data that are patient identifiable, will be stored separately from the non-identifiable data when they are no longer required for linking or quality checking. All data will be strictly handled in accordance with the Data Protection Act and NHS Code of Confidentiality. All data analyses will be done on fully anonymised data.

10.4 Archiving

To enable monitoring, peer review and audits from Health Authorities, the Investigator must agree to keep records; including the identity of all participating subjects (sufficient information to link records e.g. CRFs, blood results and GP notes), all original signed Informed Consent Forms, signed original blood data, copies of all CRFs and detailed records of drug destruction.

It is the responsibility of the Site Principal Investigator(s) to ensure all essential trial documentation and source documents (e.g. signed Informed Consent Forms, Investigator Site Files, Pharmacy Files, participants' hospital notes, copies of CRFs etc.) at their site are securely retained for 20 years The PC-CRTU trials team will be responsible for arranging the archiving of the TMF. No documents will be destroyed without prior approval from the Sponsor.

11. QUALITY CONTROL AND QUALITY ASSURANCE

11.1 Site Set-up and Initiation

All participating Investigators will be asked to supply a current CV to PC-CRTU. All members of the site research team will also be required to sign the Site Signature and Delegation Log. Prior to commencing recruitment all sites will undergo a process of site initiation. Key members of the site research team will be required to attend either a meeting or a teleconference covering aspects of the trial design, protocol procedures, Adverse Event reporting, collection and reporting of data and record keeping. Sites will be provided with an Investigator Site File and a Pharmacy File containing essential documentation, instructions, and other documentation required for the conduct of the trial. The PC-CRTU trials team must be informed immediately of any change in the site research team.

11.2 Monitoring

11.2.1 Onsite Monitoring

Monitoring will be carried out as required following a risk assessment and as documented in the monitoring plan. Any monitoring activities will be reported to the trials team and any issues noted will be followed up to resolution. Additional on-site monitoring visits may be triggered, for example by poor CRF return, poor data quality, low SAE reporting rates, excessive number of participant withdrawals or deviations. If a monitoring visit is required the Trials team will contact the site to arrange a date for the proposed visit and will provide the site with written confirmation. Investigators will allow the IMPRESS-AF monitor to access to source documents as requested. The monitoring will be conducted by PC-CRTU.

Investigators will allow the trial monitors access to source documents as requested. If a monitoring visit is required the PC-CRTU will contact the site to arrange a date for the proposed visit. Data to be verified will include:

- Informed Consent
- Eligibility
- Adverse Events

Any serious breach highlighted will be forwarded to the Trial Manager and the Sponsor as soon as possible. The sponsor will then notify the licensing authority in writing of any serious breaches of:

- The conditions and principles of GCP
- The protocol

The Sponsor must notify the licensing authority within 7 days of becoming aware of the breach.

11.2.2 Central Monitoring

Trials staff will be in regular contact with the site research team to check on progress and address any queries that they may have (by phone/fax/ email/letter). Trials staff will check incoming Case Report Forms for compliance with the protocol, data consistency, missing data and timing.

Monitoring will be done according to the PC-CRTU Monitoring SOP and the Monitoring Plan.

The Trial Coordinator will be in regular contact with the site personnel (by phone/fax/ email/letter) to check on progress and answer any queries that they may have. Trial staff will check incoming CRFs for compliance with the protocol, consistent data, missing data, timing and Investigator sign-off.

11.3 Audit and Inspection

The Investigator will permit trial-related monitoring, audits, ethical review, and regulatory inspection(s) at their site, providing direct access to source data/documents. The investigator will comply with these visits and any required follow up. Sites are also requested to notify PC-CRTU of any MHRA inspections.

11.4 Notification of Serious Breaches

In accordance with Regulation 29A of the Medicines for Human Use (Clinical Trials) Regulations 2004 and its amendments the Sponsor will notify the licensing authority in writing of any serious breach of:

- The conditions and principles of GCP in connection with that trial or;
- The protocol relating to that trial (within 7 days of becoming aware of that breach)
- For the purposes of this regulation, a "serious breach" is a breach which is likely to effect to a significant degree:
- The safety or physical or mental integrity of the subjects of the trial; or
- The scientific value of the trial

Sites are therefore requested to notify PC-CRTU who will be responsible for notifying the RGT and CRCT UoB of any suspected trial-related serious breach of GCP and/or the trial protocol. Where PC-CRTU is investigating whether or not a serious breach has occurred sites are also requested to cooperate with PC-CRTU in providing sufficient information to report the breach to the MHRA where required and in undertaking any corrective and/or preventive action.

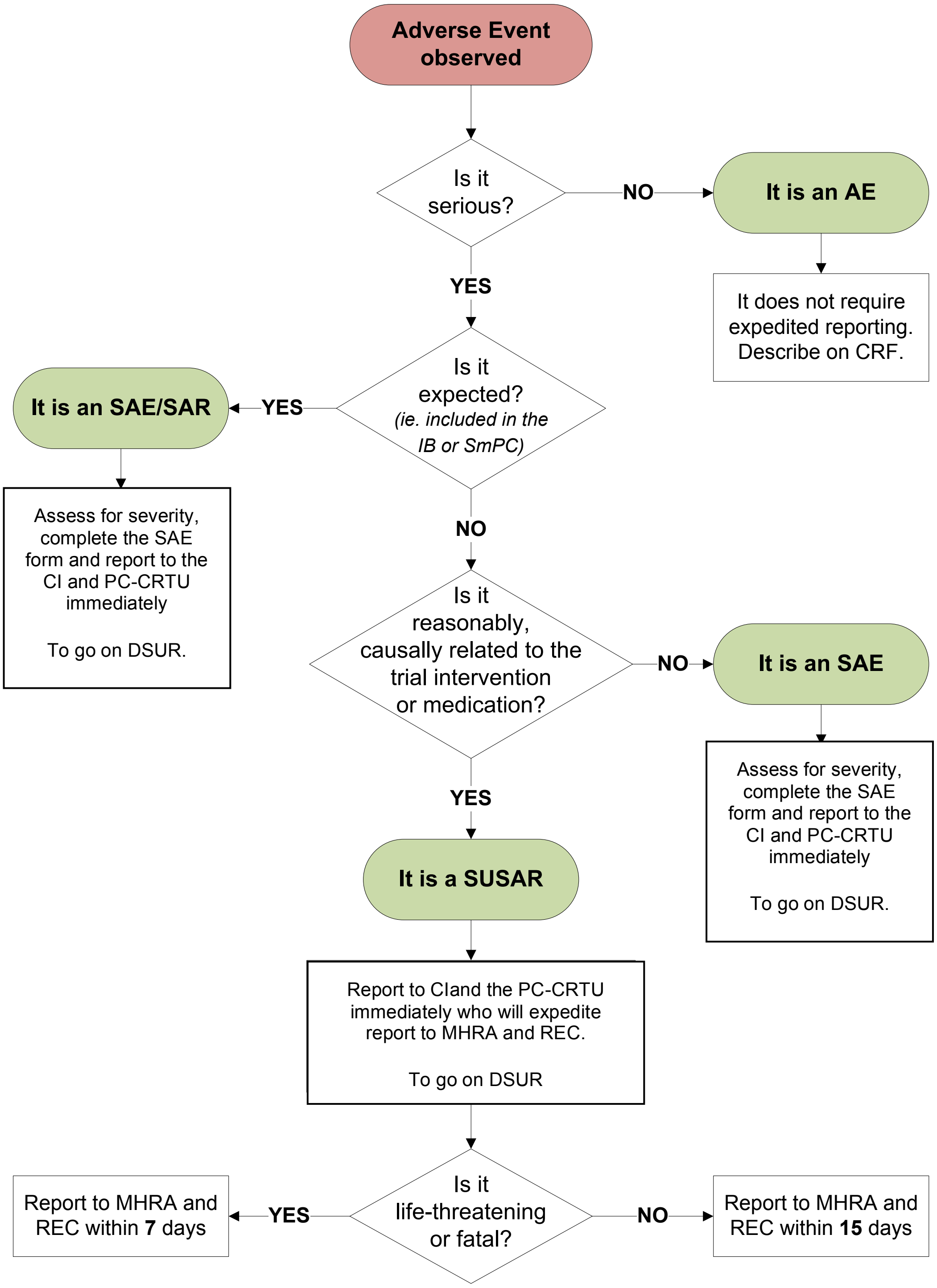
Adverse Event observed
Is it serious?
NO
It is an AE
It does not require expedited reporting. Describe on CRF.
YES
Is it expected? (ie. included in the IB or SmPC)
YES
It is an SAE/SAR
Assess for severity, complete the SAE form and report to the CI and PC-CRTU immediately
To go on DSUR.
NO
Is it reasonably, causally related to the trial intervention or medication?
NO
It is an SAE
Assess for severity, complete the SAE form and report to the CI and PC-CRTU immediately
To go on DSUR.
YES
It is a SUSAR
Report to CIand the PC-CRTU immediately who will expedite report to MHRA and REC.
To go on DSUR
Is it life-threatening or fatal?
YES
Report to MHRA and REC within 7 days
NO
Report to MHRA and REC within 15 days

12. END OF TRIAL DEFINITION

The end of trial will be the last visit of the last patient. Sufficient time will be allowed for the completion of protocol procedures, data collection and data input. The PC-CRTU trial team will notify the MHRA, main REC and RGT that the trial has ended within 90 days of the end of trial and will provide them with a summary of the clinical trial report within 12 months of the end of trial.

13. STATISTICAL CONSIDERATIONS

13.1 Analysis of Outcome Measures

Analysis will follow intention to treat principles (ITT) Following data cleaning, levels of demographics gathered at primary screening will be quantified using basic statistics (mean, SD, Median, IQR) and graphical presentations (boxplots, histograms, scatter plots) Sub division will be made by intervention /control and stratum. Likewise levels of primary and secondary outcomes at baseline will be similarly reported. Prior to any substantial analysis, preliminary impression of trends in longitudinal measurements will be provided graphically, separately for intervention and control participants.

1. Linear mixed model analysis will be used to compare peak VO_2 at two years between the intervention and the control group Covariates will be baseline peak VO_2, age, gender systolic/diastolic blood pressure and BMI measured at baseline. GP practices or recruitment centres will be included as random effects to enable findings from this trial to be judged in the context of a different group of GP practices

2. Further exploratory analyses will incorporate interaction terms between intervention/control and each of the covariates. Backward elimination will be used to remove non significant interactions and then non significant main effects. If residuals from mixed model analysis violate the assumption of a normal distribution as tested by Kolmogorov Smirnov test then transformation of the dependent variable or bootstrap analysis will be conducted. Transformations considered will be those in the Box Cox family i.e. power and log. Bootstrap analysis will involve sampling with replacement 1000 sets of data from the original data, each of the same size as the original dataset Each set will then be analysed in the same manner as the original dataset This will enable an empirical distribution of each test statistic to be formed and relocated at its null hypothesis value Significance of the statistics in the original data will then be determined from the appropriate tail area of the empirical null distribution.

Secondary analyses will also use linear or non linear mixed modelling as above but with dependent variable the secondary end points mentioned in the earlier Trial End Points section Interactions between intervention/control, and age and gender will also be included in the mixed modelling analyses to see whether differences in secondary endpoints between intervention and control participants vary with these two factors. Each mixed model analysis will be run additionally separately on each stratum. The primary QoL analysis will be the change in the overall QoL score between treatment groups over the two year duration. This will include baseline measures as a covariet. Full anaylsis will therefore be completed at the 24 months period and this will form the principle QoL analysis. The 12 month QoL assessments will allow a preliminary analysis to be undertaken to determine if changes occur within the first 12 months. These repeated measures will allow further exploratory analysis of changes in QoL to be undertaken to explore which domains are driving the changes in the QoL scores.

3. Missing values will be substituted using a multiple imputation procedure. Because of the likelihood of non normality the method of Hussain will be used.[81] This replaces the data by its Normal scores, imputes using these transformed data and then uses a polynomial relationship between Normal scores and untransformed data to give a complete set of data. This is then repeated 10 times and variation seen between repetitions and within are used to estimate the uncertainty in the imputed mean value.

In the case of missing data due to death of the participant during the trial a comparative analysis will be conducted between surviving participants and the deceased. If no difference is apparent then MI will be performed on all of the data but otherwise a complete case analysis will be conducted and a report compiled setting out the difference between the deceased and the surviving participants.

13.1.1 Power Calculations

We will send invitations to 1280 potential participants from 64 GP practices with the aim of recruiting 640 for further testing. 250 of the screened participants will then be randomised. In addition, suitable participants from outpatient clinics run by Professor Lip and Professor Kirchhoff will also be invited to take part.

Our primary outcome will be based on a ‘gold’ standard test of exercise tolerance defined by peak VO_2 using CPET and assessed at baseline and after the treatment with the trial drug. We hypothesise that the spironolactone will improve peak VO2 compared to the placebo treated subjects. We base our power calculation for peak VO2 on the published values of peak VO2 in subjects with HFpEF (16±5 ml/min/kg).[82] We anticipate a difference of 2ml/min/kg in the improvement in peak VO_2 after 2-year treatment with spironolactone compared with the control group. Unfortunately this publication does not give a standard deviation on the change in peak VO_2 from baseline but a similar trial, Edelmann et al[83] provides that statistic (5 ml/min/kg) and also reports a similar magnitude of effect.

A sample size of 100 patients in each group will have 80% power to detect a difference in means of 2.000 assuming that the common standard deviation is 5.000 (effect size =0.40) using a two group t-test with a 0.05 two-sided significance level. Sample size calculation was conducted using the nQuery software. Approximately, for 80% power at 5% significance, sample size in each group would be 15.68/(effect size)^2. To accommodate a loss to follow-up of 20% of patients from each group would imply initially recruiting a total sample size of 250 (250x.8=200) in order to still retain 200 patients in all. Loss of 20% of patients would still enable effects in excess of 2.3ml/min/kg (effect size=0.46) to be detected with 90% power at the 5% significance level. Sample size=21.0/ (effect size)^2 for 90% power, 5% significance. At the other extreme zero loss to follow up from 250 patients would lead to 88% power to detect a 2 ml/min/kg at 5% significance Prediction of 20% loss to follow up is based on a 10% loss per year and composed of 5% due to death and 5% due to other reasons. The relevant population of patients would be under regular follow up and as such would have relatively lower expected mortality.

Considering the power available for secondary endpoints, Conraads et al[84] (the ELANDD trial) give sufficient statistical information to investigate this issue for 6 minute walk distance. Comparing the difference in change from baseline between a control group (n=59) and an intervention group (n=57) using nebivolol (26m difference), gave a t statistic p value of .004. This combination of t statistic and difference between groups implies an SD on change from baseline of 48m and a power of 96% to detect a 26m difference with a sample size of 100 in each group. With our larger sample size (n=100) in each group, a smaller difference of around 18m between the two groups could be detected with 80% power for 5% significance.

Returning to Edelmann et al they supply a 95% confidence interval on the difference between two groups in change from baseline for E/e’. This confidence interval (-4.3 to -2.1) together with the mean difference (-3.2) allows estimation of the SD for change from baseline in E/e’. (SD=2.08) In turn that enables a power calculation to be performed which reveals in excess of 99% power to detect a -3.2 difference in change from baseline between intervention and control group with our sample size of 100 in each group.

From the same paper and following the same steps (95% confidence interval -11 to 1 and mean difference -5) leads to an SD of 11.35 for change from baseline) In turn that suggests 87% power for MLWHFQ showing at 5% significance a difference in change from baseline of 5 units.

As well as influencing power, sample size influences precision of estimation. The 95% confidence intervals in the table indicate the precision of the mean endpoint (difference between intervention and control in change from baseline to two years). None of the confidence intervals contain zero indicating a significant effect of the intervention

Endpoint	Mean endpoint	SD endpoint	SE endpoint	Lower 95%CI	Upper 95%CI
Peak VO_2	2	5.00	0.71	0.61	3.39
6MWTD	26	48.00	6.79	12.70	39.30
E/e'	-3.2	2.08	0.29	-3.78	-2.62
MLWHFQ	5	11.35	1.61	1.85	8.15

Based on published data in similar populations treated according to current practice the annual rate of all cause hospital admissions ranges from 20% to 45%.[16, 85] For the power calculation we used a more conservative estimate of ~20% annual admission rate The suggested conservative admission rate would allow detecting any significant reduction ≥15% in all-cause admissions in the treatment group vs. placebo group with 80% power and 5% significance. For comparing two rates R_1 and R_2 an approximate formula for sample size n would be: $n=7.84 \times (R_1+R_2)/(R_1-R_2)^2$.

Sample size – calculations and conclusions

We estimate that a sample size of 100 participants in each arm would give power of at least 80% to detect differences in primary and secondary endpoints of a magnitude consistent with published results from similar studies. Inclusion of provision for a 20% drop out rate could potentially lead to powers of near 90% or more if the assumption of a drop rate of 20% was too pessimistic.

14. TRIAL ORGANISATIONAL STRUCTURE

14.1 Sponsor

The Univeristy of Birmingham are the sponsors of this trial.

14.2 Coordinating Centre

The day-to-day management of the trial will be coordinated by the Primary Care Clinical Research and Trials Unit (PC-CRTU, fully registered by the NIHR as a trials unit) at the University of Birmingham.

14.3 Trial Management Group

The Trial *Management Group* (CI, PI, trial team, members of PC-CRTU) will meet regularly (usually monthly, with additional meetings as required) to ensure successful implementation of the trial. They will monitor participant recruitment; any departure from the expected recruitment rate will be dealt with according to the specific issues discovered. For example, GPs may need clarification of inclusion criteria and we will visit the practices to improve performance.

The Chief Investigator (CI) takes overall responsibility for the conduct of the trial. The Principal Investigator (PI) will take responsibility for all activity conducted at site. Any delegated responsibility will be documented on the site delegation log. It is the PI's responsibility to ensure that staff are appropriately trained to perform the tasks delegated to them, and that training is documented and the delegation log completed.

The research team comprises a group of experts in particular fields closely related to the project core, such as world leading clinical authorities in AF and HF (Prof Lip [CI] and Prof Kirchhof [co-investigator]), a postdoctoral clinician-expert in human molecular biology related to the project field, with extensive experience in clinical and research echocardiography (Dr Shantsila [co-investigator and PI at site]), an expert in primary care and heart failure (Dr Gill), quality of life and trials (Prof Calvert), a trial statistician (Dr Haque), an expert in exercise testing (Dr Fisher), and a participant and public involvement representative with extensive experience (Dr Mostafa, Doctor in Physics).

14.3.1 Trial Steering Committee

A Trial Steering Committee (TSC) will be appointed and will be responsible for overseeing the progress of the trial, in particular: trial progress, protocol compliance, participant safety and review of updated information. A *Steering Group* will meet every 6 months to provide support to the trial team, unless there is a specific reason to delay.

**TSC members**	
Professor Diana Adrienne Gorog, *Visiting Professor in the University of Hertfordshire; Clinical Director for Cardiac Services and Consultant Cardiologist*	Address: East and North Hertfordshire NHS Trust, Lister hospital, Coreys Mill Lane, Stevenage, SG1 4AB<br>Email: d.gorog@nhs.net; Tel: 01707 224382
Dr *Andrew Appelboam, Consultant Cardiologist*	Address: Royal Devon and Exeter Hospital (Wonford), Barrack Road, Exeter, Devon, EX2 5DW<br>Email: andy.appelboam@nhs.net;<br>Tel: 0139 241 1611
Professor Gregory YH Lip (CI), *Professor of Cardiovascular Medicine*	Address: Centre for Cardiovascular Sciences, City Hospital Birmingham, B18 7QH<br>Email: g.y.h.lip@bham.ac.uk;<br>Tel: 0121 5075080;<br>Fax: 0 121 554 4083
Professor Paulus Kirchhof, *Chair in Cardiovascular Medicine*	Address: School of Clinical and Experimental, Medicine College of Medical and Dental Sciences, University of Birmingham, Edgbaston, Birmingham, B15 2TT<br>Email: p.kirchhof@bham.ac.uk;<br>Tel: 0121 414 7042
Dr Eduard Shantsila (PI), *Clinical Postdoctoral Research Fellow in Cardiology*	Address: Centre for Cardiovascular Sciences, City Hospital Birmingham, B18 7QH<br>Email: g.y.h.lip@bham.ac.uk;<br>Tel: 0121 507 5080;<br>Fax: 0121 554 4083
A consumer representative: Mrs Caroline Renton	Email: c.renton@nhs.net

14.3.2 Data Monitoring and Ethics Committee

Please see section 1.27 for information on the structure and responsibility of the independent DMEC.

14.4 Finance

This is a clinician-initiated and clinician-led trial funded by the NIHR (National Institute for Health Research).

15. ETHICAL CONSIDERATIONS

The trial will be performed in accordance with the recommendations guiding physicians in biomedical research involving human subjects, adopted by the 18th World Medical Association General Assembly, Helsinki, Finland, June 1964, amended at the 48th World Medical Association General Assembly, Somerset West, Republic of South Africa, October 1996 (website: http://www.wma.net/en/30publications/10policies/b3/index.html).

The trial will be conducted in accordance with the Research Governance Framework for Health and Social Care, The Medicines for Human Use (Clinical Trials) Regulations and subsequent amendments and the Data Protection Act 1998 *and Human Tissue Act 2008.* This trial will be carried out under a Clinical Trial Authorisation The protocol will be submitted to and approved by the main Research Ethics Committee (REC) prior to circulation.

Before any participants are enrolled into the trial, the Principal Investigator at each site is required to obtain local R&D approval/assurance. Sites will not be permitted to enrol participants until written confirmation of R&D approval/assurance is received by the PC-CRTU trials team.

It is the responsibility of the Principal Investigator to ensure that all subsequent amendments gain the necessary local approval. This does not affect the individual clinicians' responsibility to take immediate action if thought necessary to protect the health and interest of individual participants.

16. CONFIDENTIALITY AND DATA PROTECTION

Personal data recorded on all documents will be regarded as strictly confidential and will be handled and stored in accordance with the Data Protection Act 1998.

Participants will always be identified using only their unique anonymised participant number on the Case Report Form and correspondence between PC-CRTU and the participating site.

The Investigator must maintain documents not for submission to PC-CRTU (e.g. Participant Identification Logs) in strict confidence. In the case of specific issues and/or queries from the regulatory authorities, it will be necessary to have access to the complete trial records, provided that participant confidentiality is protected.

The trial team will maintain the confidentiality of all participants' data and will not disclose information by which participants may be identified to any third party without prior consent from the participant. Representatives of the IMPRESS-AF trial team, regulatory authorities and sponsor may be required to have access to participant's notes for quality assurance purposes but participants should be reassured that their confidentiality will be respected at all times.

17. INSURANCE AND INDEMNITY

University of Birmingham employees are indemnified by the University insurers for negligent harm caused by the design or co-ordination of the clinical trials they undertake whilst in the University's employment.

In terms of liability at a site, NHS Trust and non-Trust hospitals have a duty to care for participants treated, whether or not the participant is taking part in a clinical trial. Compensation is therefore available via NHS indemnity in the event of clinical negligence having been proven.

The University of Birmingham offers indemnity for non-negligent harm. The University of Birmingham is independent of any pharmaceutical company, and as such it is not covered by the Association of the British Pharmaceutical Industry (ABPI) guidelines for participant compensation.

18. PUBLICATION POLICY

Results of this trial will be submitted for publication in a peer reviewed journal. The manuscript will be prepared by Dr E. Shantsila and authorship will be determined by mutual agreement. This will include all co-investigators providing that they meet authorship criteria plus any other authors by agreement.

Any secondary publications and presentations prepared by Investigators must be reviewed by the CI. Manuscripts must be submitted to the NIHR. Authors must acknowledge that the trial was performed with the support of sponsor (*University of Birmingham) and the NIHR.* Intellectual property rights will be addressed in the *"Clinical Trial Site Agreement"* between Sponsor and site.

19. REFERENCE LIST

1. McMurray JJ, Adamopoulos S, Anker SD, Auricchio A, Bohm M, Dickstein K, Falk V, Filippatos G, Fonseca C, Gomez-Sanchez MA, Jaarsma T, Kober L, Lip GY, Maggioni AP, Parkhomenko A, Pieske BM, Popescu BA, Ronnevik PK, Rutten FH, Schwitter J, Seferovic P, Stepinska J, Trindade PT, Voors AA, Zannad F and Zeiher A. ESC Guidelines for the diagnosis and treatment of acute and chronic heart failure 2012: The Task Force for the Diagnosis and Treatment of Acute and Chronic Heart Failure 2012 of the European Society of Cardiology. Developed in collaboration with the Heart Failure Association (HFA) of the ESC. *Eur Heart J*. 2012;33:1787-847.

2. Tsang TS, Barnes ME, Gersh BJ, Bailey KR and Seward JB. Risks for atrial fibrillation and congestive heart failure in patients >/=65 years of age with abnormal left ventricular diastolic relaxation. *Am J Cardiol*. 2004;93:54-8.

3. Tsang TS, Gersh BJ, Appleton CP, Tajik AJ, Barnes ME, Bailey KR, Oh JK, Leibson C, Montgomery SC and Seward JB. Left ventricular diastolic dysfunction as a predictor of the first diagnosed nonvalvular atrial fibrillation in 840 elderly men and women. *J Am Coll Cardiol*. 2002;40:1636-44.

4. Fonarow GC, Stough WG, Abraham WT, Albert NM, Gheorghiade M, Greenberg BH, O'Connor CM, Sun JL, Yancy CW and Young JB. Characteristics, treatments, and outcomes of patients with preserved systolic function hospitalized for heart failure: a report from the OPTIMIZE-HF Registry. *J Am Coll Cardiol*. 2007;50:768-77.

5. Paulus WJ and van Ballegoij JJ. Treatment of heart failure with normal ejection fraction: an inconvenient truth! *J Am Coll Cardiol*. 2010;55:526-37.

6. Hobbs FD, Fitzmaurice DA, Mant J, Murray E, Jowett S, Bryan S, Raftery J, Davies M and Lip G. A randomised controlled trial and cost-effectiveness study of systematic screening (targeted and total population screening) versus routine practice for the detection of atrial fibrillation in people aged 65 and over. The SAFE study. *Health Technol Assess*. 2005;9:iii-iv, ix-x, 1-74.

7. Fitzmaurice DA, Hobbs FD, Jowett S, Mant J, Murray ET, Holder R, Raftery JP, Bryan S, Davies M, Lip GY and Allan TF. Screening versus routine practice in detection of atrial fibrillation in patients aged 65 or over: cluster randomised controlled trial. *BMJ*. 2007;335:383.

8. Kannel WB, Wolf PA, Benjamin EJ and Levy D. Prevalence, incidence, prognosis, and predisposing conditions for atrial fibrillation: population-based estimates. *Am J Cardiol*. 1998;82:2N-9N.

9. Nieuwlaat R, Eurlings LW, Cleland JG, Cobbe SM, Vardas PE, Capucci A, Lopez-Sendon JL, Meeder JG, Pinto YM and Crijns HJ. Atrial fibrillation and heart failure in cardiology practice: reciprocal impact and combined management from the perspective of atrial fibrillation: results of the Euro Heart Survey on atrial fibrillation. *J Am Coll Cardiol*. 2009;53:1690-8.

10. Lip GY, Golding DJ, Nazir M, Beevers DG, Child DL and Fletcher RI. A survey of atrial fibrillation in general practice: the West Birmingham Atrial Fibrillation Project. *Br J Gen Pract*. 1997;47:285-9.

11. Zarifis J, Beevers G and Lip GY. Acute admissions with atrial fibrillation in a British multiracial hospital population. *Br J Clin Pract*. 1997;51:91-6.

12. Lip GY, Tean KN and Dunn FG. Treatment of atrial fibrillation in a district general hospital. *Br Heart J*. 1994;71:92-5.

13. Cowie MR, Wood DA, Coats AJ, Thompson SG, Suresh V, Poole-Wilson PA and Sutton GC. Survival of patients with a new diagnosis of heart failure: a population based study. *Heart*. 2000;83:505-10.

14. Hobbs FD, Roalfe AK, Davis RC, Davies MK and Hare R. Prognosis of all-cause heart failure and borderline left ventricular systolic dysfunction: 5 year mortality follow-up of the Echocardiographic Heart of England Screening Study (ECHOES). *Eur Heart J*. 2007;28:1128-34.

15. http://www.nice.org.uk/nicemedia/live/13099/50514/50514.pdf.

16. Linssen GC, Rienstra M, Jaarsma T, Voors AA, van Gelder IC, Hillege HL and van Veldhuisen DJ. Clinical and prognostic effects of atrial fibrillation in heart failure patients with reduced and preserved left ventricular ejection fraction. *Eur J Heart Fail*. 2011;13:1111-20.

17. Olsson LG, Swedberg K, Ducharme A, Granger CB, Michelson EL, McMurray JJ, Puu M, Yusuf S and Pfeffer MA. Atrial fibrillation and risk of clinical events in chronic heart failure with and without left ventricular systolic dysfunction: results from the Candesartan in Heart failure-Assessment of Reduction in Mortality and morbidity (CHARM) program. *J Am Coll Cardiol*. 2006;47:1997-2004.

18. McKelvie RS, Komajda M, McMurray J, Zile M, Ptaszynska A, Donovan M, Carson P and Massie BM. Baseline plasma NT-proBNP and clinical characteristics: results from the irbesartan in heart failure with preserved ejection fraction trial. *J Card Fail*. 2010;16:128-34.

19. Fung JW, Sanderson JE, Yip GW, Zhang Q and Yu CM. Impact of atrial fibrillation in heart failure with normal ejection fraction: a clinical and echocardiographic study. *J Card Fail*. 2007;13:649-55.

20. McManus DD, Hsu G, Sung SH, Saczynski JS, Smith DH, Magid DJ, Gurwitz JH, Goldberg RJ and Go AS. Atrial fibrillation and outcomes in heart failure with preserved versus reduced left ventricular ejection fraction. *J Am Heart Assoc*. 2013;2:e005694.

21. Zile MR, Baicu CF and Gaasch WH. Diastolic heart failure--abnormalities in active relaxation and passive stiffness of the left ventricle. *N Engl J Med*. 2004;350:1953-9.

22. Westermann D, Kasner M, Steendijk P, Spillmann F, Riad A, Weitmann K, Hoffmann W, Poller W, Pauschinger M, Schultheiss HP and Tschope C. Role of left ventricular stiffness in heart failure with normal ejection fraction. *Circulation*. 2008;117:2051-60.

23. McMurray JJ, Adamopoulos S, Anker SD, Auricchio A, Bohm M, Dickstein K, Falk V, Filippatos G, Fonseca C, Gomez-Sanchez MA, Jaarsma T, Kober L, Lip GY, Maggioni AP, Parkhomenko A, Pieske BM, Popescu BA, Ronnevik PK, Rutten FH, Schwitter J, Seferovic P, Stepinska J, Trindade PT, Voors AA, Zannad F, Zeiher A, Bax JJ, Baumgartner H, Ceconi C, Dean V, Deaton C, Fagard R, Funck-Brentano C, Hasdai D, Hoes A, Kirchhof P, Knuuti J, Kolh P, McDonagh T, Moulin C, Reiner Z, Sechtem U, Sirnes PA, Tendera M, Torbicki A, Vahanian A, Windecker S, Bonet LA, Avraamides P, Ben Lamin HA, Brignole M, Coca A, Cowburn P, Dargie H, Elliott P, Flachskampf FA, Guida GF, Hardman S, Iung B, Merkely B, Mueller C, Nanas JN, Nielsen OW, Orn S, Parissis JT and Ponikowski P. ESC Guidelines for the diagnosis and treatment of acute and chronic heart failure 2012: The Task Force for the Diagnosis and Treatment of Acute and Chronic Heart Failure 2012 of the European Society of Cardiology. Developed in collaboration with the Heart Failure Association (HFA) of the ESC. *Eur Heart J*. 2012;33:1787-847.

24. Redfield MM, Jacobsen SJ, Borlaug BA, Rodeheffer RJ and Kass DA. Age- and gender-related ventricular-vascular stiffening: a community-based study. *Circulation*. 2005;112:2254-62.

25. Lam CS, Roger VL, Rodeheffer RJ, Bursi F, Borlaug BA, Ommen SR, Kass DA and Redfield MM. Cardiac structure and ventricular-vascular function in persons with heart failure and preserved ejection fraction from Olmsted County, Minnesota. *Circulation*. 2007;115:1982-90.

26. Kitzman DW, Higginbotham MB, Cobb FR, Sheikh KH and Sullivan MJ. Exercise intolerance in patients with heart failure and preserved left ventricular systolic function: failure of the Frank-Starling mechanism. *J Am Coll Cardiol*. 1991;17:1065-72.

27. Ahmed SH, Clark LL, Pennington WR, Webb CS, Bonnema DD, Leonardi AH, McClure CD, Spinale FG and Zile MR. Matrix metalloproteinases/tissue inhibitors of metalloproteinases: relationship between changes in proteolytic determinants of matrix composition and structural, functional, and clinical manifestations of hypertensive heart disease. *Circulation*. 2006;113:2089-96.

28. Martos R, Baugh J, Ledwidge M, O'Loughlin C, Conlon C, Patle A, Donnelly SC and McDonald K. Diastolic heart failure: evidence of increased myocardial collagen turnover linked to diastolic dysfunction. *Circulation*. 2007;115:888-95.

29. Schotten U, Verheule S, Kirchhof P and Goette A. Pathophysiological mechanisms of atrial fibrillation: a translational appraisal. *Physiol Rev*. 2011;91:265-325.

30. Daccarett M, Badger TJ, Akoum N, Burgon NS, Mahnkopf C, Vergara G, Kholmovski E, McGann CJ, Parker D, Brachmann J, Macleod RS and Marrouche NF. Association of left atrial fibrosis detected by delayed-enhancement magnetic resonance imaging and the risk of stroke in patients with atrial fibrillation. *J Am Coll Cardiol*. 2011;57:831-8.

31. Shantsila E, Shantsila A, Blann AD and Lip GY. Left ventricular fibrosis in atrial fibrillation. *Am J Cardiol*. 2013;111:996-1001.

32. Weber KT. Aldosterone in congestive heart failure. *N Engl J Med*. 2001;345:1689-97.

33. Burniston JG, Saini A, Tan LB and Goldspink DF. Aldosterone induces myocyte apoptosis in the heart and skeletal muscles of rats in vivo. *J Mol Cell Cardiol*. 2005;39:395-9.

34. Brilla CG, Zhou G, Matsubara L and Weber KT. Collagen metabolism in cultured adult rat cardiac fibroblasts: response to angiotensin II and aldosterone. *J Mol Cell Cardiol*. 1994;26:809-20.

35. Zannad F, Alla F, Dousset B, Perez A and Pitt B. Limitation of excessive extracellular matrix turnover may contribute to survival benefit of spironolactone therapy in patients with congestive heart failure: insights from the randomized aldactone evaluation study (RALES). Rales Investigators. *Circulation*. 2000;102:2700-6.

36. Weber KT. The proinflammatory heart failure phenotype: a case of integrative physiology. *Am J Med Sci*. 2005;330:219-26.

37. Tsai CT, Chiang FT, Tseng CD, Hwang JJ, Kuo KT, Wu CK, Yu CC, Wang YC, Lai LP and Lin JL. Increased expression of mineralocorticoid receptor in human atrial fibrillation and a cellular model of atrial fibrillation. *J Am Coll Cardiol*. 2010;55:758-70.

38. Endemann DH, Touyz RM, Iglarz M, Savoia C and Schiffrin EL. Eplerenone prevents salt-induced vascular remodeling and cardiac fibrosis in stroke-prone spontaneously hypertensive rats. *Hypertension*. 2004;43:1252-7.

39. Deswal A, Richardson P, Bozkurt B and Mann DL. Results of the Randomized Aldosterone Antagonism in Heart Failure With Preserved Ejection Fraction Trial (RAAM-PEF). *J Card Fail*. 2011;17:634-42.

40. Izawa H, Murohara T, Nagata K, Isobe S, Asano H, Amano T, Ichihara S, Kato T, Ohshima S, Murase Y, Iino S, Obata K, Noda A, Okumura K and Yokota M. Mineralocorticoid receptor antagonism ameliorates left ventricular diastolic dysfunction and myocardial fibrosis in mildly symptomatic patients with idiopathic dilated cardiomyopathy: a pilot study. *Circulation*. 2005;112:2940-5.

41. Edwards NC, Ferro CJ, Kirkwood H, Chue CD, Young AA, Stewart PM, Steeds RP and Townend JN. Effect of spironolactone on left ventricular systolic and diastolic function in patients with early stage chronic kidney disease. *Am J Cardiol*. 2010;106:1505-11.

42. Edwards NC, Steeds RP, Stewart PM, Ferro CJ and Townend JN. Effect of spironolactone on left ventricular mass and aortic stiffness in early-stage chronic kidney disease: a randomized controlled trial. *J Am Coll Cardiol*. 2009;54:505-12.

43. Edelmann F, Wachter R, Schmidt AG, Kraigher-Krainer E, Colantonio C, Kamke W, Duvinage A, Stahrenberg R, Durstewitz K, Loffler M, Dungen HD, Tschope C, Herrmann-Lingen C, Halle M, Hasenfuss G, Gelbrich G and Pieske B. Effect of spironolactone on diastolic function and exercise capacity in patients with heart failure with preserved ejection fraction: the Aldo-DHF randomized controlled trial. *JAMA*. 2013;309:781-91.

44. Edelmann F, Schmidt AG, Gelbrich G, Binder L, Herrmann-Lingen C, Halle M, Hasenfuss G, Wachter R and Pieske B. Rationale and design of the 'aldosterone receptor blockade in diastolic heart failure' trial: a double-blind, randomized, placebo-controlled, parallel group study to determine the effects of spironolactone on exercise capacity and diastolic function in patients with symptomatic diastolic heart failure (Aldo-DHF). *Eur J Heart Fail*. 2010;12:874-82.

45. Shah SJ, Heitner JF, Sweitzer NK, Anand IS, Kim HY, Harty B, Boineau R, Clausell N, Desai AS, Diaz R, Fleg JL, Gordeev I, Lewis EF, Markov V, O'Meara E, Kobulia B, Shaburishvili T, Solomon SD, Pitt B, Pfeffer MA and Li R. Baseline characteristics of patients in the treatment of preserved cardiac function heart failure with an aldosterone antagonist trial. *Circ Heart Fail*. 2013;6:184-92.

46. Desai AS, Lewis EF, Li R, Solomon SD, Assmann SF, Boineau R, Clausell N, Diaz R, Fleg JL, Gordeev I, McKinlay S, O'Meara E, Shaburishvili T, Pitt B and Pfeffer MA. Rationale and design of the treatment of preserved cardiac function heart failure with an aldosterone antagonist trial: a randomized, controlled study of spironolactone in patients with symptomatic heart failure and preserved ejection fraction. *Am Heart J*. 2011;162:966-972 e10.

47. Mahadevan G, Davis RC, Frenneaux MP, Hobbs FD, Lip GY, Sanderson JE and Davies MK. Left ventricular ejection fraction: are the revised cut-off points for defining systolic dysfunction sufficiently evidence based? *Heart*. 2008;94:426-8.

48. Pate RR, Pratt M, Blair SN, Haskell WL, Macera CA, Bouchard C, Buchner D, Ettinger W, Heath GW, King AC and et al. Physical activity and public health. A recommendation from the Centers for Disease Control and Prevention and the American College of Sports Medicine. *JAMA*. 1995;273:402-7.

49. Albouaini K, Egred M, Alahmar A and Wright DJ. Cardiopulmonary exercise testing and its application. *Heart*. 2007;93:1285-92.

50. Arena R, Guazzi M, Myers J, Chase P, Bensimhon D, Cahalin LP, Peberdy MA, Ashley E, West E and Forman DE. The prognostic utility of cardiopulmonary exercise testing stands the test of time in patients with heart failure. *J Cardiopulm Rehabil Prev*. 2012;32:198-202.

51. Cahalin LP, Chase P, Arena R, Myers J, Bensimhon D, Peberdy MA, Ashley E, West E, Forman DE, Pinkstaff S, Lavie CJ and Guazzi M. A meta-analysis of the prognostic significance of cardiopulmonary exercise testing in patients with heart failure. *Heart Fail Rev*. 2012.

52. Gibbons RJ, Balady GJ, Beasley JW, Bricker JT, Duvernoy WF, Froelicher VF, Mark DB, Marwick TH, McCallister BD, Thompson PD, Winters WL, Jr., Yanowitz FG, Ritchie JL, Cheitlin MD, Eagle KA, Gardner TJ, Garson A, Jr., Lewis RP, O'Rourke RA and Ryan TJ. ACC/AHA guidelines for exercise testing: executive summary. A report of the American College of Cardiology/American Heart Association Task Force on Practice Guidelines (Committee on Exercise Testing). *Circulation*. 1997;96:345-54.

53. Deftereos S, Giannopoulos G, Kossyvakis C, Raisakis K, Theodorakis A, Kaoukis A, Toli K, Panagopoulou V, Driva M, Mantas I, Pyrgakis V and Alpert MA. Short-term fluctuations of plasma NT-proBNP levels in patients with new-onset atrial fibrillation: a way to assess time of onset? *Heart*. 2010;96:1033-6.

54. Camm AJ, Kirchhof P, Lip GY, Schotten U, Savelieva I, Ernst S, Van Gelder IC, Al-Attar N, Hindricks G, Prendergast B, Heidbuchel H, Alfieri O, Angelini A, Atar D, Colonna P, De Caterina R, De Sutter J, Goette A, Gorenek B, Heldal M, Hohloser SH, Kolh P, Le Heuzey JY, Ponikowski P and Rutten FH. Guidelines for the management of atrial fibrillation: the Task Force for the Management of Atrial Fibrillation of the European Society of Cardiology (ESC). *Eur Heart J*. 2010;31:2369-429.

55. Chung I, Goyal D, Macfadyen RJ and Lip GY. The effects of maximal treadmill graded exercise testing on haemorheological, haemodynamic and flow cytometry platelet markers in patients with systolic or diastolic heart failure. *Eur J Clin Invest*. 2008;38:150-8.

56. Fisher JP, Kim A, Young CN and Fadel PJ. Carotid baroreflex control of arterial blood pressure at rest and during dynamic exercise in aging humans. *Am J Physiol Regul Integr Comp Physiol*. 2010;299:R1241-7.

57. Fisher JP, Ogoh S, Young CN, Raven PB and Fadel PJ. Regulation of middle cerebral artery blood velocity during dynamic exercise in humans: influence of aging. *J Appl Physiol*. 2008;105:266-73.

58. Watson T, Shantsila E, Karthikeyan VJ, Jessani S, Goon PK and Lip GY. The effects of exercise stress testing, diurnal variation and temporal decline on circulating progenitor cells. *Thromb Haemost*. 2010;103:419-25.

59. Goyal D, Logie IM, Nadar SK, Lip GY and Macfadyen RJ. Generalized obesity but not that characterized by raised waist-hip ratio is associated with increased perceived breathlessness during treadmill exercise testing. *Cardiovasc Ther*. 2009;27:10-6.

60. Rector TS, Carson PE, Anand IS, McMurray JJ, Zile MR, McKelvie RS, Komajda M, Kuskowski M and Massie BM. Assessment of long-term effects of irbesartan on heart failure with preserved ejection fraction as measured by the minnesota living with heart failure questionnaire in the irbesartan in heart failure with preserved systolic function (I-PRESERVE) trial. *Circ Heart Fail*. 2012;5:217-25.

61. Rector TS, Kubo SH and Cohn JN. Validity of the Minnesota Living with Heart Failure questionnaire as a measure of therapeutic response to enalapril or placebo. *Am J Cardiol*. 1993;71:1106-7.

62. Rector TS and Cohn JN. Assessment of patient outcome with the Minnesota Living with Heart Failure questionnaire: reliability and validity during a randomized, double-blind, placebo-controlled trial of pimobendan. Pimobendan Multicenter Research Group. *Am Heart J*. 1992;124:1017-25.

63. Brooks R. EuroQol: the current state of play. *Health Policy*. 1996;37:53-72.

64. Rabin R and de Charro F. EQ-5D: a measure of health status from the EuroQol Group. *Ann Med*. 2001;33:337-43.

65. Mahadevan G, Dwivedi G, Williams L, Steeds RP and Frenneaux M. Epidemiology and diagnosis of heart failure with preserved left ventricular ejection fraction: rationale and design of the study. *Eur J Heart Fail*. 2012;14:106-12.

66. Myers J, Prakash M, Froelicher V, Do D, Partington S and Atwood JE. Exercise capacity and mortality among men referred for exercise testing. *N Engl J Med*. 2002;346:793-801.

67. Myers J, Kaykha A, George S, Abella J, Zaheer N, Lear S, Yamazaki T and Froelicher V. Fitness versus physical activity patterns in predicting mortality in men. *Am J Med*. 2004;117:912-8.

68. Scott JM, Haykowsky MJ, Eggebeen J, Morgan TM, Brubaker PH and Kitzman DW. Reliability of peak exercise testing in patients with heart failure with preserved ejection fraction. *Am J Cardiol*. 2012;110:1809-13.

69. Marburger CT, Brubaker PH, Pollock WE, Morgan TM and Kitzman DW. Reproducibility of cardiopulmonary exercise testing in elderly patients with congestive heart failure. *Am J Cardiol*. 1998;82:905-9.

70. ATS/ACCP Statement on cardiopulmonary exercise testing. *Am J Respir Crit Care Med*. 2003;167:211-77.

71. Faggiano P, D'Aloia A, Gualeni A, Brentana L and Dei Cas L. The 6 minute walking test in chronic heart failure: indications, interpretation and limitations from a review of the literature. *Eur J Heart Fail*. 2004;6:687-91.

72. Opasich C, Pinna GD, Mazza A, Febo O, Riccardi R, Riccardi PG, Capomolla S, Forni G, Cobelli F and Tavazzi L. Six-minute walking performance in patients with moderate-to-severe heart failure; is it a useful indicator in clinical practice? *Eur Heart J*. 2001;22:488-96.

73. Lang RM, Bierig M, Devereux RB, Flachskampf FA, Foster E, Pellikka PA, Picard MH, Roman MJ, Seward J, Shanewise JS, Solomon SD, Spencer KT, Sutton MS and Stewart WJ. Recommendations for chamber quantification: a report from the American Society of Echocardiography's Guidelines and Standards Committee and the Chamber Quantification Writing Group, developed in conjunction with the European Association of Echocardiography, a branch of the European Society of Cardiology. *J Am Soc Echocardiogr*. 2005;18:1440-63.

74. Kusunose K, Yamada H, Nishio S, Tomita N, Niki T, Yamaguchi K, Koshiba K, Yagi S, Taketani Y, Iwase T, Soeki T, Wakatsuki T, Akaike M and Sata M. Clinical utility of single-beat E/e' obtained by simultaneous recording of flow and tissue Doppler velocities in atrial fibrillation with preserved systolic function. *JACC Cardiovasc Imaging*. 2009;2:1147-56.

75. Nagueh SF, Appleton CP, Gillebert TC, Marino PN, Oh JK, Smiseth OA, Waggoner AD, Flachskampf FA, Pellikka PA and Evangelisa A. Recommendations for the evaluation of left ventricular diastolic function by echocardiography. *Eur J Echocardiogr*. 2009;10:165-93.

76. http://www.euroqol.org/about-eq-5d.html.

77. http://www.euroqol.org/fileadmin/user_upload/Documenten/PDF/Folders_Flyers/UserGuide_EQ-5D-5L_v2.0_October_2013.pdf.

78. Herdman M, Gudex C, Lloyd A, Janssen M, Kind P, Parkin D, Bonsel G and Badia X. Development and preliminary testing of the new five-level version of EQ-5D (EQ-5D-5L). *Qual Life Res*. 2011;20:1727-36.

79. van Hout B, Janssen MF, Feng YS, Kohlmann T, Busschbach J, Golicki D, Lloyd A, Scalone L, Kind P and Pickard AS. Interim scoring for the EQ-5D-5L: mapping the EQ-5D-5L to EQ-5D-3L value sets. *Value Health*. 2012;15:708-15.

80. Devlin NJ and Krabbe PF. The development of new research methods for the valuation of EQ-5D-5L. *Eur J Health Econ*. 2013;14 Suppl 1:S1-3.

81. Hussain S, Mohammed M, Haque M, Holder R, Macleod J and R. H. A simple method to ensure plausible multiple imputation for continuous multivariate data. *Communications in Statistics – Simulation and Computation*. 2010;39:1779-1784.

82. Cicoira M, Zanolla L, Rossi A, Golia G, Franceschini L, Brighetti G, Marino P and Zardini P. Long-term, dose-dependent effects of spironolactone on left ventricular function and exercise tolerance in patients with chronic heart failure. *J Am Coll Cardiol*. 2002;40:304-10.

83. Edelmann F, Gelbrich G, Dungen HD, Frohling S, Wachter R, Stahrenberg R, Binder L, Topper A, Lashki DJ, Schwarz S, Herrmann-Lingen C, Loffler M, Hasenfuss G, Halle M and Pieske B. Exercise training improves exercise capacity and diastolic function in patients with heart failure with preserved ejection fraction: results of the Ex-DHF (Exercise training in Diastolic Heart Failure) pilot study. *J Am Coll Cardiol*. 2011;58:1780-91.

84. Conraads VM, Metra M, Kamp O, De Keulenaer GW, Pieske B, Zamorano J, Vardas PE, Bohm M and Dei Cas L. Effects of the long-term administration of nebivolol on the clinical symptoms, exercise capacity, and left ventricular function of patients with diastolic dysfunction: results of the ELANDD study. *Eur J Heart Fail*. 2012;14:219-25.

85. Meinertz T, Kirch W, Rosin L, Pittrow D, Willich SN and Kirchhof P. Management of atrial fibrillation by primary care physicians in Germany: baseline results of the ATRIUM registry. *Clin Res Cardiol*. 2011;100:897-905.

20. ABBREVIATIONS AND DEFINITIONS:

Term	Description
**SOP**	See "Standard Operating Procedures"
**Standard Operating Procedures (SOP)**	Standard Operating Procedures are detailed written instructions to achieve uniformity in the performance of a specific function. They define tasks, allocate responsibilities, detail processes, indicate documents and templates to be used and cross-reference to other work instructions and guidance or policy documents. They are standards to which the UoB may be audited or inspected.
**Adverse Event (AE)**	Any untoward medical occurrence in a participant or clinical trial subject administered a medicinal product and which does not necessarily have a causal relationship with this treatment.
**Adverse Reaction (AR)**	All untoward and unintended responses to an IMP related to any dose administered.<br>Comment:<br>An AE judged by either the reporting Investigator or Sponsor as having causal relationship to the IMP qualifies as an AR. The expression reasonable causal relationship means to convey in general that there is evidence or argument to suggest a causal relationship.
**Serious Adverse Event (SAE)**	Any untoward medical occurrence or effect that:<br>• Results in death Is life-threatening*<br>• Requires hospitalisation or prolongation of existing inparticipants' hospitalisation<br>• Results in persistent or significant disability or incapacity<br>• Is a congenital anomaly/birth defect<br>• Or is otherwise considered medically significant by the Investigator**<br>Comments:<br>The term severe is often used to describe the intensity (severity) of a specific event. This is not the same as serious, which is based on participants/event outcome or action criteria.<br>* Life threatening in the definition of an SAE refers to an event in which the participant was at risk of death at the time of the event; it does not refer to an event that hypothetically might have caused death if it were more severe.<br>** Medical judgment should be exercised in deciding whether an AE is serious in other situations. Important AEs that are not immediately life threatening or do not result in death or hospitalisation but may jeopardise the subject or may require intervention to prevent one of the other outcomes listed in the definition above, should be considered serious.
**Serious Adverse**	An Adverse Reaction which also meets the definition of a Serious Adverse

**Reaction (SAR)**	Event
**Unexpected Adverse Reaction (UAR)**	An AR, the nature or severity of which is not consistent with the applicable product information (e.g. Investigator Brochure for an unapproved IMP or (compendium of) Summary of Product Characteristics (SPC) for a licensed product).<br>When the outcome of an AR is not consistent with the applicable product information the AR should be considered unexpected.
**Suspected Unexpected Serious Adverse Reaction (SUSAR)**	A SAR that is unexpected i.e. the nature, or severity of the event is not consistent with the applicable product information.<br>A SUSAR should meet the definition of an AR, UAR and SAR.
**Source data**	All information in original records and certified copies of original records of clinical findings, observations, or other activities in a clinical trial necessary for the reconstruction and evaluation of the trial
**PC-CRTU**	Primary Care Clinical Research and Trials Unit (University of Birmingham): The co-ordinating centre for the trial.

APPENDIX 1. Mechanistic substudy

Clinical utility of biomarkers of fibrosis and hemostasis in chronic atrial fibrillation: A substudy of the IMPRESS-AF trial

Abstract:

Experimental data suggest role of cardiac fibrosis and hemostasis in cardiac stiffening and development of heart failure with preserved ejection fraction in atrial fibrillation (AF) However limited data exist on clinical utility of blood markers of fibrosis and hemostasis in chronic AF with adequate oral anticoagulation for stroke prevention. The proposed study aims to establish potential of blood markers of fibrosis and hemostasis as predictors of deterioration of exercise tolerance, quality of life, diastolic function and rates of hospital admissions in patients with chronic AF and preserved left ventricular ejection fraction; to determine utility of the fibrotic and hemostatic biomarkers to predict capacity of aldosterone inhibitor, spironolactone to prevent deterioration of exercise tolerance, quality of life, diastolic function and prevent hospital admissions in these patients.

The double-blinded randomised IMproved exercise tolerance In heart failure With PReserved Ejection fraction by Spironolactone On myocardial fibrosiS In Atrial Fibrillation (IMPRESS-AF) trial awarded by NIHR-EME program will assess impact of 2-year treatment with an aldosterone antagonist, spironolactone vs placebo in 250 AF patients with preserved cardiac contractility. The trial will establish impact of spironolactone on exercise tolerance, quality of life, left ventricular diastolic function, and rates of hospitalisations. We propose to use population of the IMPRESS-AF patients to address the aims above using the blood tests from those patients.

Aims of the substudy: (i) To establish utility of blood markers of fibrosis and hemostasis as predictors of deterioration of exercise tolerance, quality of life, diastolic function and rates of hospital admissions in patients with chronic atrial fibrillation (AF) and preserved left ventricular (LV) ejection fraction (EF); (ii) to determine utility of the fibrotic and hemostatic biomarkers to predict capacity of aldosterone inhibitor, spironolactone to prevent deterioration of exercise tolerance, quality of life, diastolic function and prevent hospital admissions in these patients.

Original hypotheses of the proposed study:

We hypothesise that in patients with permanent AF with preserved LVEF (on standard oral anticoagulant therapy) high systemic levels of biomarkers of fibrosis and procoagulant values of hemostatic parameters can be used as predictors of deterioration of exercise tolerance, quality of life, diastolic function hospital admission and predictors of higher clinical efficacy of treatment with spironolactone.

Background: The prevalence of AF was 4.7% in general practice in people aged 65 years or more. A third of AF patients suffer symptomatic HF, of which most have HFpEF.[1] In patients with HFpEF, AF is present in about 40% and is independently associated with an increased risk of death and hospitalisation with HF.[2, 3] Data from the CHARM program, which studied 7,599 patients with symptomatic HF, demonstrated that AF was associated with increased risk of the death or hospitalisation for worsening HFpEF.[3]

Activation of profibrotic pathways, increased production of myocardial collagen lead to increased pressure load in the heart, diastolic dysfunction and increased cardiac stiffness. This process is linked to an increased myocardial collagen turnover and shift in the balance between matrix metalloproteinases (MMP) and their inhibitors in favour of excessive myocardial fibrosis.[4, 5] Published evidence from AF populations supports a central role of atrial fibrosis in electrical and structural atrial

remodelling, and an independent predictive value for the high risk of cerebrovascular events.[6] We have previously demonstrated that presence of AF and its progression from paroxysmal to chronic form are associated with incremental increase in left ventricular fibrosis, which significantly correlated with deterioration in parameters of diastolic function.[7]

Aldosterone. Aldosterone promotes cardiac fibrosis via myocardial inflammation, oxidative stress, and cardiomyocyte apoptosis and also direct stimulation of cardiac fibroblasts to express type I and III fibrillar collagen genes.[8, 9] Cardiac expression of mineralocorticoid receptors is increased in AF, thus augmenting the genomic effects of aldosterone.[10] Aldosterone antagonists (i.e., spironolactone or eplerenone) ameliorate LV fibrosis in animal models.[11]

Biomarkers of collagen turnover and galectin-3. Plasma biomarkers of collagen turnover are easily accessible surrogate measures of systemic fibrotic processes. Procollagen secreted by fibroblasts undergoes enzymatic cleavage of its end-terminal sequences to enable collagen fibre formation. Serum levels of the pro-peptides, such as amino-terminal pro-peptide of pro-collagen type I (PINP) and type III (PIIINP) correlate with the amount of fibrillar collagen deposited.[12, 13] For example, improved post-infarct LV remodelling in patients treated with spironolactone was associated with suppression of PIIINP levels.[14] Galectin-3 has recently emerged as a marker of fibrosis implicated in pathogenesis of HF. Experimental data showed that galectin-3 is directly involved in myocardial fibrogenesis and mediates aldosterone-induced vascular fibrosis.[15, 16] Clinical studies show that galectin-3 predicts incident HF in the community and it provides incremental prognostic information and predicts LV remodelling in chronic systolic HF.[17, 18]

Monocytes. Monocytes are immune cells with numerous biological functions (e.g., phagocytosis, inflammation). Monocytes have been implicated in fibrosis of different tissues.[19] Along with (myo)fibroblasts, monocyte-derived macrophages are a source of TGFβ1, the key profibrotic factor.[20] Activated monocytes represent a major MMP (matrix metalloprotease) source.[21] Indeed, monocytes/macrophages co-localise with myofibroblasts in areas of fibrosis during cardiac hypertrophy.[22] Also, monocytic cells can directly differentiate into fibroblasts and cardiac myofibroblasts.[23] Inhibition of monocyte accumulation in the myocardium suppressed myocardial fibrosis in hypertensive rats.[24] The ability of an aldosterone inhibitor to reduce myocardial fibrosis, myocyte apoptosis, and MMP activity in mice with chronic pressure overload was associated with reduced macrophage infiltration.[25] Similar results have been obtained in animal models of diastolic dysfunction.[11, 26] Additionally aldosterone leads to cardiac invasion of proinflammatory mononuclear cells.[27]

Whilst experimental data support role of cardiac fibrosis in development of HFpEF and role of monocytes in profibrotic processes little data exist on clinical utility of blood markers of fibrosis and monocyte profibrotic characteristics in chronic AF.

Hemostatic parameters (thromboelastography, micro-plate assay, microparticles and platelet funciton). Our previouse data showed significant association between hemostatic paramentes and microparticles and monocyte characteristics in patients with acute coronary syndromes and HF with reduced ejection fraction. However scarce data are available on role of these parameters in AF with HFpEF. Other laboratory tools for determining the rate of thrombus formation and fibrinolysis in whole blood and plasma include the thrombelastograph and the micro-plate assay, respectively.[28, 29] We also have a well validated protocol for analysis of different types of plasma microparticles.

Reduced nitric oxide availability are present in patients with HF as well as patients with new onset AF.[30-32] Platelets isolated from HF patients present reduced intracellular cGMP levels as compared to healthy volunteers, resulting in reduced phosphorylation of the cGMP-dependent protein kinase substrate VASP.[33] Platelet activation is tightly regulated by activatory and inhibitory signals, but in

cardiovascular disease this balance is often shifted to a pro-aggregatory state. Since cyclic nucleotides represent the main activation-dampening signal in platelets, impairment of cGMP signalling leads to decreased platelet inhibition, hyper aggregability and increased thrombotic risk.

It is now established that under certain conditions inorganic nitrate and nitrite may serve as a source of nitric oxide alternative to endothelial production in the vasculature and other tissues.[34, 35] Platelets are exposed to circulatory nitrite, and this could represent a source of nitric oxide and an alternative mechanism of control of platelet activation when endothelial dysfunction is present.

Nitrite is under consideration as a therapeutic agent. We have very recently demonstrated that short term nitrite infusion improves cardiac function in HF patients.[36] Contrarily to the use of organic nitrates, which is subject to the development of tolerance thus limiting their beneficial effects, treatment with nitrite does not induce this phenomenon.[37-39]

Our studies and others' currently indicate that nitrite exerts inhibitory effects in platelets but only at supraphysiological concentrations (100 mM-1mM, Borgognone et al. unpublished data) or in the presence of other blood cells.[40, 41] While the nature of the mechanism of action of nitrite in platelets is unclear, it has been demonstrated in a mouse model that when endothelial function is disrupted by deletion of endothelial nitric oxide synthase, nitrite becomes relevant as a source of nitric oxide to inhibit platelet function.[42]

In our study on the arteriolar effects of infused nitrite we have observed hyper dilatation in the vasculature of HF patients compared to healthy volunteers.[39] Although the mechanistic basis of this increased sensitivity to nitrite in HF patients remains unclear, it is possible that, where nitric oxide availability of endothelial origin is reduced, nitrite has a more important role as a vascular source of nitric oxide, and platelets might be inhibited by lower concentrations of nitrite. Moreover, if nitrite were to be used as a therapeutic agent, the circulating concentrations of this mediator would substantially exceed the physiological level, with greater effects on platelet function.

Study protocol

A mechanistic substudy was originally planned to be a part of the IMPRESS-AF clinical trial. However the NIHR requested to reduce the study costs and we had to remove this part from the original application. However we were encouraged by the NIHR to seek additional funding for the mechanistic (biomarker) part of the study. We plan to fulfil this through this proposed biomarkers analysis in the IMPRESS-AF study population. The study will use blood samples routinely collected in IMPRESS-AF trial patients during the study visits (40 ml each): (i) at the time of patient randomisation, and (ii) at the time of the final visit after the completion of the randomised clinical trial (IMPRESS-AF). Statistical analyses will be performed utilising clinical data from the IMPRESS-AF trial.

Methods: (i) Plasma markers of fibrosis/collagen turnover (PICP and PIIICP) and galectin-3 will be measured using high quality commercially available enzyme-linked immunosorbent assay (ELISA) kits (please seen cost justification section of specifics of the kits).

(ii) Quantification of blood monocyte subsets in fresh blood will be done by flow cytometry using the BD FACSCalibur flow cytometer (Becton Dickinson, Oxford, UK [BD]) as previously published by our group.[43-45] Monocyte subsets will be defined as CD14++CD16–CCR2+ cells ('classical'), CD14++CD16+CCR2+ cells ('intermediate') and CD14+CD16++CCR2- cells ('non-classical').[43, 46] Absolute counts of monocyte subsets (cells/µl) will be obtained by calculating the number of monocytes proportional to the number of count beads according to the manufacturer's recommendations.

(iii) Assessment of profibrotic monocyte activation will be performed in 'classical' CD14++CD16– monocytes, which represent ~85% of all monocytes and were related to negative outcomes in HFpEF and MMP production. Isolation of this subset will be performed according to the protocol established in the department.[43] Peripheral blood mononuclear cells will be isolated by Ficoll-Paque density gradient centrifugation, from 15ml of anticoagulated blood. Monocytes will be further isolated by magnetic sorting using VarioMACS immunomagnetic sorter and Biotec Monocyte Isolation Kit II (both from Miltenyi Biotec, Dusseldorf, Germany). The protocol allows achievement of >90% monocyte purity. Total RNA and protein will be extracted from each monocyte sample using TRIzol® reagent (Invitrogen, Carlsbad, CA, USA), according to the manufacturer's instructions and stored at -80 ºC, until use. Real-time reverse transcription (RT-PCR) will be used to measure monocyte expression of MMP1, MMP9, MMP13, TGFβ1 in batched analyses.

(iv) Thromboelastography and micro-plate assay. Citrated venous blood will be analysed for indices of thrombogenesis and fibrinolysis using a throboelastography analyser within 2 hours of collection according to the manufacturer's advice. Thrombus formation and autolysis will be monitored for 60 minutes after the addition of calcium to whole blood supplemented with kaolin. For the micro-plate assay, citrated plasma will used.[28] Micro-plate assay will be comprised of two parts. Firstly, in a thrombogenesis assay, 25µl plasma, 75µl TRIS-NaCl buffer, and 50µl thrombin will be added to the wells of 96-well microtitre plate. Clot formation will be followed for 30 minutes at 37°C in a micro-titre plate reader by change in optical density. Clot dissolution promoted by exogenous tissue plasminogen activator will be followed for 30 minutes at 37°C in a microplate reader by change in optical density.

(v) Isolation of platelets. 18 ml of venous blood will be drawn in 3.8% sodium citrate (final concentration) for platelet studies (aggregation and western blot). Whole blood will be added 1:10 v/v ACD (sodium citrate25 g/l, glucose 20 g/l, citric acid 15 g/l) and centrifuged at 200 x g for 20 min. Platelet rich plasma (PRP) will be collected up to 0.5 cm from the interface with the RBC pellet in order to minimise RBCs contamination. Platelets will be isolated from PRP by centrifugation at 1000 x g for 10 min following addition of PGI2 (0.1µg/ml; to inhibit platelet activation). The resulting platelet pellet will be re-suspended in Tyrode's buffer (134mM NaCl, 0.34mM, Na2HPO4, 2.9mM KCl, 12mM NaHCO3, 20mM HEPES,5mM glucose, 1mM MgCl2, pH 7.3) and centrifuged at 1000 x g in the presence of 0.1µg/ml PGI2 and 1:10 v/v ACD. The supernatant will be discarded and the platelet pellet will be re-suspended in Tyrode's buffer at 2 x 10^{8}/ml for aggregation studies and at 5 x 10^{8}/ml for western blot sample preparation. The washed platelet suspensions will be allowed to rest for 1 h prior to experimentation to allow the effects of PGI2 to decay.

(vi) Determination of nitrite basal plasma concentration. 4 ml of venous blood will be drawn into an EDTA tube containing NEM (10 mM final concentration) for determination of basal levels of circulating nitrite. The tube will be immediately spun at 1000 x g for 15 min. Plasma will be collected and immediately stored at -80C. The samples will be shipped to Prof. M. Feelisch at the University of Southampton for determination of nitrate/nitrite basal concentration.

(vii) Platelet aggregation and dense granule secretion. Washed platelets, suspended at 2x10^{8}/ml, will be analysed by light transmission aggregation (LTA) and dense granule secretion using a lumi-dual aggregometer (model 460VS; Chronolog, Labmedics, Manchester, UK) under continuous stirring at 1200 rpm. The optical density of the platelet suspension will be measured against a blank reading of Tyrode's buffer (100%). Chronolume (Chronolog, Labmedics, Manchester, UK) will be incubated in the suspension 1: 30 for measurement of ATP secretion from dense granules. Calibration will be obtained by addition of 1.2 nmol ATP at the end of the reaction. Sodium nitrite (NaNO2) will be incubated at concentrations ranging from 1 to 1000 mM 5 minutes before platelet activation with 3 mg/ml collagen. Aggregation will be recorded for 5 minutes. Depending on the availability of

platelets, additional reactions will be performed with the soluble guanylate cyclase inhibitor ODQ or nitric oxide scavengers such as PTIO and OxyHb in combination with NaNO2.

(viii) Western blot. Washed platelets, suspended at $5x10^8$/ml, will be incubated with NaNO2 as for platelet aggregation studies. Incubations will be stopped by adding 5x reducing sample buffer after 5 minutes. Samples will be boiled for 5 min and spun prior to SDS-PAGE (10%) and transfer onto a PVDF membrane. Immunoblotting will be performed for the detection of p-Ser239 and p-Ser157 VASP and analysed by band densitometry.

Sample size – calculations: The sample size has been originally calculated to provide sufficient power to assess impact of the spironolactone treatment for the outcomes of the IMPRESS-AF trial and it will provide statistical power sufficient to assess utility of the fibrotic parameters to predict the study outcomes and efficacy of treatment with spironolactone (with more 80% power) using appropriate regression analysis (linear regression and Cox regression). Further exploratory analyses will incorporate interaction terms between intervention/control and each of the covariates, including parameters of fibrosis. Backward elimination will be used to remove non-significant interactions and then non-significant main effects.

Regarding the nitrite/platelet part of the study we aim to obtain pilot data for a future bigger and specifically designed study. Based on our previous studies it is estimated that n=20 AF patients will be necessary for the evaluation of plasma nitrite concentrations.[39] For platelet aggregation studies, previous studies utilised n=14 HF patients for the determination of the effect of GTN in whole blood.[47] Based on the above, we estimate that n=40 HF patients will be sufficient to determine the effects of nitrite on aggregation and at molecular level in isolated platelets in comparison to healthy controls (n=20 for platelet aggregation, n=20 for western blot).

References:

1. Nieuwlaat R, Eurlings LW, Cleland JG, Cobbe SM, Vardas PE, Capucci A, Lopez-Sendon JL, Meeder JG, Pinto YM and Crijns HJ. Atrial fibrillation and heart failure in cardiology practice: reciprocal impact and combined management from the perspective of atrial fibrillation: results of the Euro Heart Survey on atrial fibrillation. *J Am Coll Cardiol*. 2009;53:1690-8.

2. Linssen GC, Rienstra M, Jaarsma T, Voors AA, van Gelder IC, Hillege HL and van Veldhuisen DJ. Clinical and prognostic effects of atrial fibrillation in heart failure patients with reduced and preserved left ventricular ejection fraction. *European journal of heart failure*. 2011;13:1111-20.

3. Olsson LG, Swedberg K, Ducharme A, Granger CB, Michelson EL, McMurray JJ, Puu M, Yusuf S and Pfeffer MA. Atrial fibrillation and risk of clinical events in chronic heart failure with and without left ventricular systolic dysfunction: results from the Candesartan in Heart failure-Assessment of Reduction in Mortality and morbidity (CHARM) program. *J Am Coll Cardiol*. 2006;47:1997-2004.

4. Ahmed SH, Clark LL, Pennington WR, Webb CS, Bonnema DD, Leonardi AH, McClure CD, Spinale FG and Zile MR. Matrix metalloproteinases/tissue inhibitors of metalloproteinases: relationship between changes in proteolytic determinants of matrix composition and structural, functional, and clinical manifestations of hypertensive heart disease. *Circulation*. 2006;113:2089-96.

5. Martos R, Baugh J, Ledwidge M, O'Loughlin C, Conlon C, Patle A, Donnelly SC and McDonald K. Diastolic heart failure: evidence of increased myocardial collagen turnover linked to diastolic dysfunction. *Circulation*. 2007;115:888-95.

6. Schotten U, Verheule S, Kirchhof P and Goette A. Pathophysiological mechanisms of atrial fibrillation: a translational appraisal. *Physiol Rev*. 2011;91:265-325.

7. Shantsila E, Shantsila A, Blann AD and Lip GY. Left ventricular fibrosis in atrial fibrillation. *Am J Cardiol*. 2013;111:996-1001.

8. Burniston JG, Saini A, Tan LB and Goldspink DF. Aldosterone induces myocyte apoptosis in the heart and skeletal muscles of rats in vivo. *J Mol Cell Cardiol*. 2005;39:395-9.

9. Brilla CG, Zhou G, Matsubara L and Weber KT. Collagen metabolism in cultured adult rat cardiac fibroblasts: response to angiotensin II and aldosterone. *J Mol Cell Cardiol*. 1994;26:809-20.

10. Tsai CT, Chiang FT, Tseng CD, Hwang JJ, Kuo KT, Wu CK, Yu CC, Wang YC, Lai LP and Lin JL. Increased expression of mineralocorticoid receptor in human atrial fibrillation and a cellular model of atrial fibrillation. *J Am Coll Cardiol*. 2010;55:758-70.

11. Endemann DH, Touyz RM, Iglarz M, Savoia C and Schiffrin EL. Eplerenone prevents salt-induced vascular remodeling and cardiac fibrosis in stroke-prone spontaneously hypertensive rats. *Hypertension*. 2004;43:1252-7.

12. Querejeta R, Varo N, Lopez B, Larman M, Artinano E, Etayo JC, Martinez Ubago JL, Gutierrez-Stampa M, Emparanza JI, Gil MJ, Monreal I, Mindan JP and Diez J. Serum carboxy-terminal propeptide of procollagen type I is a marker of myocardial fibrosis in hypertensive heart disease. *Circulation*. 2000;101:1729-35.

13. Gonzalez A, Lopez B, Ravassa S, Beaumont J, Arias T, Hermida N, Zudaire A and Diez J. Biochemical markers of myocardial remodelling in hypertensive heart disease. *Cardiovasc Res*. 2009;81:509-18.

14. Hayashi M, Tsutamoto T, Wada A, Tsutsui T, Ishii C, Ohno K, Fujii M, Taniguchi A, Hamatani T, Nozato Y, Kataoka K, Morigami N, Ohnishi M, Kinoshita M and Horie M. Immediate administration of mineralocorticoid receptor antagonist spironolactone prevents post-infarct left ventricular remodeling associated with suppression of a marker of myocardial collagen synthesis in patients with first anterior acute myocardial infarction. *Circulation*. 2003;107:2559-65.

15. Yu L, Ruifrok WP, Meissner M, Bos EM, van Goor H, Sanjabi B, van der Harst P, Pitt B, Goldstein IJ, Koerts JA, van Veldhuisen DJ, Bank RA, van Gilst WH, Sillje HH and de Boer RA. Genetic and pharmacological inhibition of galectin-3 prevents cardiac remodeling by interfering with myocardial fibrogenesis. *Circ Heart Fail*. 2013;6:107-17.

16. Calvier L, Miana M, Reboul P, Cachofeiro V, Martinez-Martinez E, de Boer RA, Poirier F, Lacolley P, Zannad F, Rossignol P and Lopez-Andres N. Galectin-3 mediates aldosterone-induced vascular fibrosis. *Arterioscler Thromb Vasc Biol*. 2013;33:67-75.

17. Ho JE, Liu C, Lyass A, Courchesne P, Pencina MJ, Vasan RS, Larson MG and Levy D. Galectin-3, a marker of cardiac fibrosis, predicts incident heart failure in the community. *Journal of the American College of Cardiology*. 2012;60:1249-56.

18. Motiwala SR, Szymonifka J, Belcher A, Weiner RB, Baggish AL, Sluss P, Gaggin HK, Bhardwaj A and Januzzi JL. Serial measurement of galectin-3 in patients with chronic heart failure: results from the ProBNP Outpatient Tailored Chronic Heart Failure Therapy (PROTECT) study. *Eur J Heart Fail*. 2013;15:1157-63.

19. Karlmark KR, Weiskirchen R, Zimmermann HW, Gassler N, Ginhoux F, Weber C, Merad M, Luedde T, Trautwein C and Tacke F. Hepatic recruitment of the inflammatory Gr1+ monocyte subset upon liver injury promotes hepatic fibrosis. *Hepatology*. 2009;50:261-74.

20. Riemann D, Wollert HG, Menschikowski J, Mittenzwei S and Langner J. Immunophenotype of lymphocytes in pericardial fluid from patients with different forms of heart disease. *Int Arch Allergy Immunol*. 1994;104:48-56.

21. Newby AC. Metalloproteinase expression in monocytes and macrophages and its relationship to atherosclerotic plaque instability. *Arterioscler Thromb Vasc Biol*. 2008;28:2108-14.

22. Hinglais N, Heudes D, Nicoletti A, Mandet C, Laurent M, Bariety J and Michel JB. Colocalization of myocardial fibrosis and inflammatory cells in rats. *Lab Invest*. 1994;70:286-94.

23. Labat ML, Bringuier AF, Arys-Philippart C, Arys A and Wellens F. Monocytic origin of fibrosis. In vitro transformation of HLA-DR monocytes into neo-fibroblasts: inhibitory effect of all-trans retinoic acid on this process. *Biomed Pharmacother*. 1994;48:103-11.

24. Ishimaru K, Ueno H, Kagitani S, Takabayashi D, Takata M and Inoue H. Fasudil attenuates myocardial fibrosis in association with inhibition of monocyte/macrophage infiltration in the heart of DOCA/salt hypertensive rats. *J Cardiovasc Pharmacol*. 2007;50:187-94.

25. Kuster GM, Kotlyar E, Rude MK, Siwik DA, Liao R, Colucci WS and Sam F. Mineralocorticoid receptor inhibition ameliorates the transition to myocardial failure and decreases oxidative stress and inflammation in mice with chronic pressure overload. *Circulation*. 2005;111:420-7.

26. Cittadini A, Monti MG, Isgaard J, Casaburi C, Stromer H, Di Gianni A, Serpico R, Saldamarco L, Vanasia M and Sacca L. Aldosterone receptor blockade improves left ventricular remodeling and increases ventricular fibrillation threshold in experimental heart failure. *Cardiovasc Res*. 2003;58:555-64.

27. Weber KT. The proinflammatory heart failure phenotype: a case of integrative physiology. *Am J Med Sci*. 2005;330:219-26.

28. Ranjit P, Lau Y, Lip GY and Blann AD. Development and validation of a new assay for assessing clot integrity. *Vascular pharmacology*. 2015;71:102-7.

29. Luddington RJ. Thrombelastography/thromboelastometry. *Clinical and laboratory haematology*. 2005;27:81-90.

30. Marti CN, Gheorghiade M, Kalogeropoulos AP, Georgiopoulou VV, Quyyumi AA and Butler J. Endothelial dysfunction, arterial stiffness, and heart failure. *Journal of the American College of Cardiology*. 2012;60:1455-69.

31. Shantsila E, Wrigley BJ, Blann AD, Gill PS and Lip GY. A contemporary view on endothelial function in heart failure. *European journal of heart failure*. 2012;14:873-81.

32. Procter NE, Ball J, Liu S, Hurst N, Nooney VB, Goh V, Stafford I, Heresztyn T, Carrington M, Ngo DT, Hylek EM, Isenberg JS, Chirkov YY, Stewart S, Horowitz JD and Investigators S. Impaired platelet nitric oxide response in patients with new onset atrial fibrillation. *International journal of cardiology*. 2015;179:160-5.

33. Shah A, Passacquale G, Gkaliagkousi E, Ritter J and Ferro A. Platelet nitric oxide signalling in heart failure: role of oxidative stress. *Cardiovascular research*. 2011;91:625-31.

34. Bailey JC, Feelisch M, Horowitz JD, Frenneaux MP and Madhani M. Pharmacology and therapeutic role of inorganic nitrite and nitrate in vasodilatation. *Pharmacol Ther*. 2014;144:303-20.

35. Lundberg JO, Weitzberg E and Gladwin MT. The nitrate-nitrite-nitric oxide pathway in physiology and therapeutics. *Nat Rev Drug Discov*. 2008;7:156-67.

36. Ormerod JO, Arif S, Mukadam M, Evans JD, Beadle R, Fernandez BO, Bonser RS, Feelisch M, Madhani M and Frenneaux MP. Short-term intravenous sodium nitrite infusion improves cardiac and pulmonary hemodynamics in heart failure patients. *Circulation Heart failure*. 2015;8:565-71.

37. Munzel T, Daiber A and Gori T. More answers to the still unresolved question of nitrate tolerance. *European heart journal*. 2013;34:2666-73.

38. Dejam A, Hunter CJ, Tremonti C, Pluta RM, Hon YY, Grimes G, Partovi K, Pelletier MM, Oldfield EH, Cannon RO, 3rd, Schechter AN and Gladwin MT. Nitrite infusion in humans and nonhuman primates: endocrine effects, pharmacokinetics, and tolerance formation. *Circulation*. 2007;116:1821-31.

39. Maher AR, Arif S, Madhani M, Abozguia K, Ahmed I, Fernandez BO, Feelisch M, O'Sullivan AG, Christopoulos A, Sverdlov AL, Ngo D, Dautov R, James PE, Horowitz JD and Frenneaux MP. Impact of chronic congestive heart failure on pharmacokinetics and vasomotor effects of infused nitrite. *Br J Pharmacol*. 2013;169:659-70.

40. Huang Z, Shiva S, Kim-Shapiro DB, Patel RP, Ringwood LA, Irby CE, Huang KT, Ho C, Hogg N, Schechter AN and Gladwin MT. Enzymatic function of hemoglobin as a nitrite reductase that produces NO under allosteric control. *The Journal of clinical investigation*. 2005;115:2099-107.

41. Srihirun S, Sriwantana T, Unchern S, Kittikool D, Noulsri E, Pattanapanyasat K, Fucharoen S, Piknova B, Schechter AN and Sibmooh N. Platelet inhibition by nitrite is dependent on erythrocytes and deoxygenation. *PloS one*. 2012;7:e30380.

42. Apostoli GL, Solomon A, Smallwood MJ, Winyard PG and Emerson M. Role of inorganic nitrate and nitrite in driving nitric oxide-cGMP-mediated inhibition of platelet aggregation in vitro and in vivo. *Journal of thrombosis and haemostasis : JTH*. 2014;12:1880-9.

43. Shantsila E, Wrigley B, Tapp L, Apostolakis S, Montoro-Garcia S, Drayson MT and Lip GY. Immunophenotypic characterization of human monocyte subsets: possible implications for cardiovascular disease pathophysiology. *J Thromb Haemost*. 2011;9:1056-66.

44. Tapp LD, Shantsila E, Wrigley BJ, Pamukcu B and Lip GY. The CD14++CD16+ monocyte subset and monocyte-platelet interactions in patients with ST-elevation myocardial infarction. *J Thromb Haemost*. 2012;10:1231-41.

45. Wrigley BJ, Shantsila E, Tapp LD and Lip GY. CD14++CD16+ monocytes in patients with acute ischaemic heart failure. *Eur J Clin Invest*. 2013;43:121-30.

46. Ziegler-Heitbrock L, Ancuta P, Crowe S, Dalod M, Grau V, Hart DN, Leenen PJ, Liu YJ, MacPherson G, Randolph GJ, Scherberich J, Schmitz J, Shortman K, Sozzani S, Strobl H, Zembala M, Austyn JM and Lutz MB. Nomenclature of monocytes and dendritic cells in blood. *Blood*. 2010;116:e74-80.

47. Chirkov YY, De Sciscio M, Sverdlov AL, Leslie S, Sage PR and Horowitz JD. Hydralazine does not ameliorate nitric oxide resistance in chronic heart failure. *Cardiovascular drugs and therapy / sponsored by the International Society of Cardiovascular Pharmacotherapy*. 2010;24:131-7.
